# *Leishmania braziliensis* enhances monocyte responses to promote anti-tumor activity

**DOI:** 10.1016/j.celrep.2024.113932

**Published:** 2024-03-07

**Authors:** Jéssica Cristina dos Santos, María Moreno, Lisa U. Teufel, Sofía Chilibroste, Samuel T. Keating, Laszlo Groh, Jorge Domínguez-Andrés, David L. Williams, Zuchao Ma, Douglas W. Lowman, Harry E. Ensley, Boris Novakovic, Fátima Ribeiro-Dias, Mihai G. Netea, José A. Chabalgoity, Leo A.B. Joosten

**Affiliations:** 1 Department of Internal Medicine and Radboud Center of Infectious Diseases (RCI), Radboud University Medical Center, Nijmegen, the Netherlands; 2 Laboratory for Vaccine Research, Departamento de Desarrollo Biotecnológico, Instituto de Higiene, Facultad de Medicina, Universidad de la República, Montevideo, Uruguay; 3 Department of Surgery, Quillen College of Medicine, Center of Excellence in Inflammation, Infectious Disease and Immunity, East Tennessee State University, Johnson City, TN, USA; 4 Murdoch Children’s Research Institute and Department of Pediatrics, University of Melbourne, Melbourne, VIC, Australia; 5 Instituto de Patologia Tropical e Saúde Pública, Universidade Federal de Goiás, Goiênia, Goiás, Brazil; 6 Department for Immunology and Metabolism, Life and Medical Sciences Institute (LIMES), University of Bonn, Bonn, Germany; 7 Department of Medical Genetics, Iuliu Hatieganu University of Medicine and Pharmacy, Cluj-Napoca, Romania; 8 Lead contact

## Abstract

Innate immune cells can undergo long-term functional reprogramming after certain infections, a process called trained immunity (TI). Here, we focus on antigens of *Leishmania braziliensis*, which induced anti-tumor effects via trained immunity in human monocytes. We reveal that monocytes exposed to promastigote antigens of *L. braziliensis* develop an enhanced response to subsequent exposure to Toll-like receptor (TLR)2 or TLR4 ligands. Mechanistically, the induction of TI in monocytes by *L. braziliensis* is mediated by multiple pattern recognition receptors, changes in metabolism, and increased deposition of H3K4me3 at the promoter regions of immune genes. The administration of *L. braziliensis* exerts potent anti-tumor capabilities by delaying tumor growth and prolonging survival of mice with non-Hodgkin lymphoma. Our work reveals mechanisms of TI induced by *L. braziliensis in vitro* and identifies its potential for cancer immunotherapy.

## In brief

dos Santos et al. report the ability of *Leishmania* parasites to improve the responses of monocytes through trained immunity. Trained macrophages impact tumor growth and survival of mice bearing lymphoma and melanoma, offering opportunities for the use of trained immunity in cancer therapy.

## Graphical Abstract

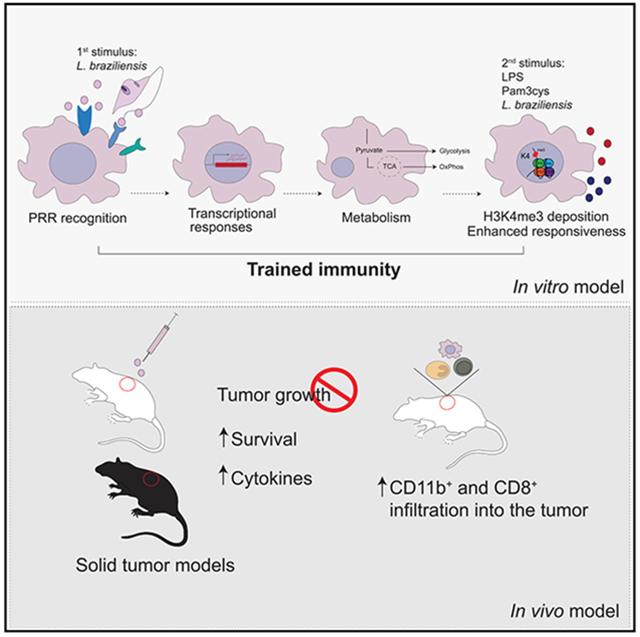

## INTRODUCTION

*Leishmania (Viannia) braziliensis* is the most prevalent species causing American tegumentary leishmaniasis (ATL) in Latin America. The spectrum of clinical manifestations of *L. braziliensis* infections ranges from self-healing cutaneous lesions to chronic ulcers and mucosal involvement.^1,2^ Macrophages are the main host cells used by the parasites for intracellular replication and long-term survival.^3^ Activation of macrophages by interferon-γ and tumor necrosis factor (TNF) contributes to the control of growth of the parasite. Nevertheless, increased numbers of macrophages in the lesion site have been associated with lesion size in early lesions and with necrosis throughout the course of the disease, suggesting a direct role for macrophages in the pathology of *L. braziliensis*-induced lesions.^4-7^

It was recently shown that monocytes and macrophages can build memory traits through previous immune exposure and develop long-term functional reprogramming via a process called trained immunity (TI). TI occurs upon the contact of monocytes to vaccines such as bacillus Calmette-Guérin vaccine (BCG) or to pathogen-associated molecular patterns and damage-associated molecular patterns, including the cell-wall component from *Candida albicans*, β-glucan, oxidized low-density lipoprotein (oxLDL), and urate, respectively.^8-11^ The increased responsiveness of trained monocytes after encountering a secondary stimulus has been associated with metabolic and epigenetic rewiring.^12,13^ Numerous metabolites that are generated as a product of changes in cellular metabolism can act as cofactors for epigenetic writers and erasers, linking immunometabolism with long-term changes in gene regulation.^14^ At the chromatin level, enrichment of trimethylation of lysine 4 of H3 histones (H3K4me3), H3K4 monomethylation (H3K4me1), and H3K27 acetylation at regulatory elements of proinflammatory cytokine genes are associated with increased transcriptional competency.^15-17^ The clinical relevance of the induction of TI has been explored in areas of atherosclerosis, immunodeficiency, autoinflammatory diseases, non-related infections, and anti-cancer therapy.^11,18-20^

The induction of inflammatory responses by macrophages in response to *L. braziliensis* might offer pragmatic benefits for the treatment of pathologies in which immunosuppression is harmful, most notably, e.g., while treating certain cancers. Over-coming the immunosuppressive tumor microenvironment attributed mainly to the presence of tumor-associated macrophages, regulatory T cells, and myeloid-derived suppressor cells remains the major challenge to the successful application of immunotherapy.^21,22^ In the present study, we postulated that the exposure of human monocytes to *Leishmania* spp. induces functional reprogramming of monocytes and macrophages via induction of TI. In addition, we hypothesized that activation of TI by *Leishmania* may induce anti-tumor effects. Deciphering how myeloid cells are epigenetically reprogrammed for a hyperinflammatory state during *Leishmania* infections could provide a potential breakthrough not only in targeting the induction of deleterious inflammation but also in inducing anti-tumor macrophages with effector responses in the tumor microenvironment.

## RESULTS

### *Leishmania* parasites induce TI in human primary monocytes

**Figure 1.**
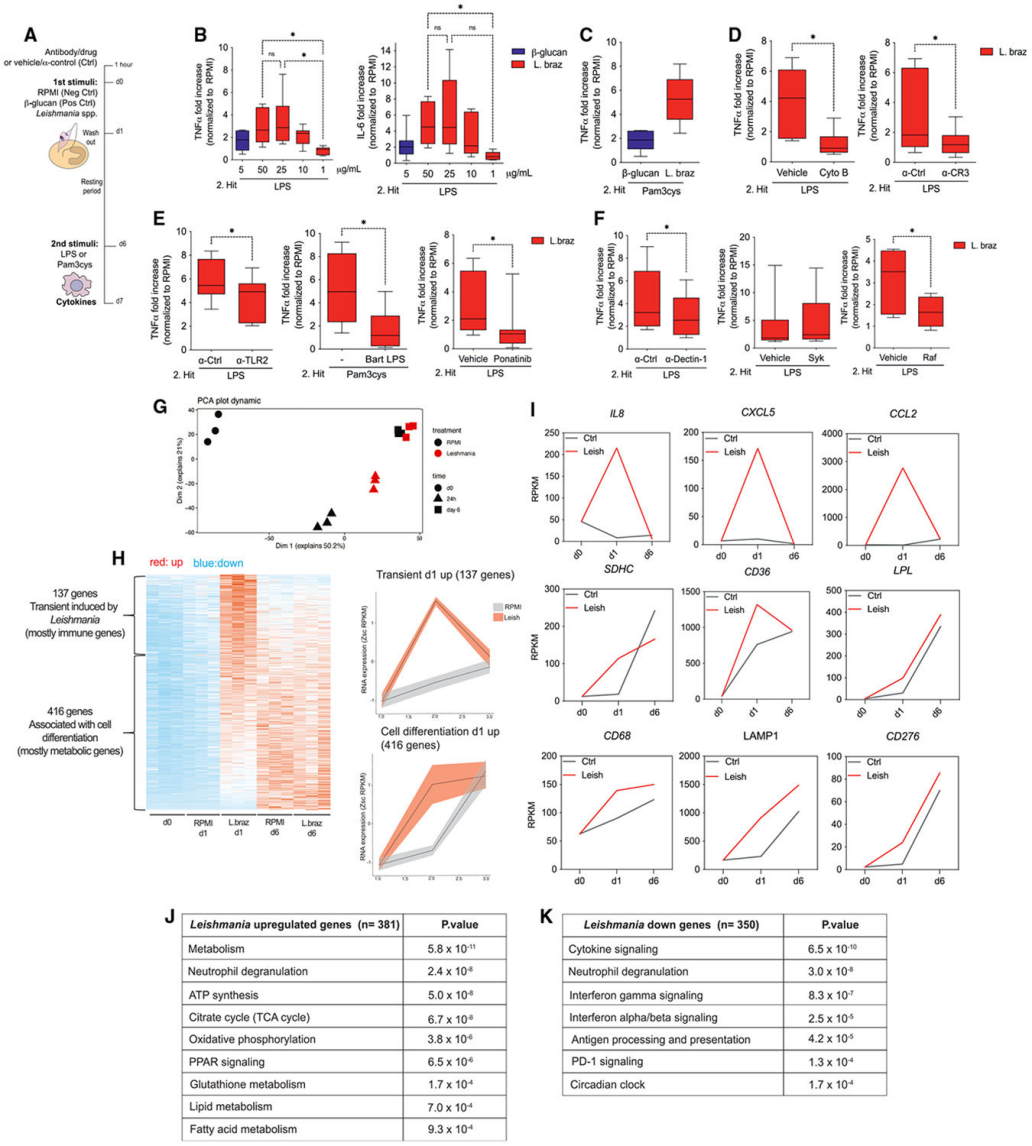
*L. braziliensis* induces trained immunity *in vitro* (A) Experimental setup. (B) TNF and IL-6 release from monocytes trained with RPMI, β-glucan (5 μg/mL), or *L. braziliensis* lysates (50, 25, 10, and 1 μg/mL) for 24 h after LPS (10 ng/mL) restimulation at day 7, measured by ELISA. (C–F) (C) TNF release from *L. braziliensis* lysates-trained macrophages (25 μg/mL) after restimulation with Pam_3_Cys (10 μg/mL) at day 7. TNF production after LPS restimulation at day 7 of *L. braziliensis*-trained (25 μg/mL) macrophages ± (D) cytochalasin B and anti-CR3, (E) anti-TLR2 and *B. quintana* LPS (Bart. LPS, TLR4 antagonist), the RIP2 kinase inhibitor ponatinib, and (F) anti-dectin-1 and the Syk and Raf inhibitors R406 and GW5074, respectively. As controls, isotype control antibody, RPMI (−), or RPMI + DMSO (vehicle) were used as indicated. n = 6 independent donors. Cytokine measurements are represented as fold increase normalized to RPMI (non-trained cells). Data in (B–F) are shown in box-and-whiskers (minimum-to-maximum) plots from two independent experiments (*p < 0.05 by Wilcoxon test; ns, p > 0.05). (G) Principal component analysis (PCA) plot of gene expression dynamic across treatment and time points (RPMI, black; *L. braziliensis* lysates, red; day 0, circles; 24 h, triangles; day 6, squares). (H) Heatmap of differentiated expressed genes (upregulated, red; downregulated, blue) at baseline (day 0) and at the indicated time points (day 1 and day 6) after RPMI or *L. braziliensis* lysates exposure (25 μg/mL). (I) Time-resolved median expression of genes transiently or gradually induced in *L. braziliensis*-exposed cells. (J and K) Most abundant pathways (J) upregulated and (K) downregulated based on gene expression analysis 24 h after treatment. n = 3 independent donors from one experiment. See also Figures S1 and S2.

Given the importance of monocytes and macrophages for *Leishmania* infections,^7,23^ we hypothesize that the exposure of human monocytes to *Leishmania* parasites induces TI. We exposed human primary monocytes to different lysate concentrations obtained from stationary-phase *L. braziliensis* promastigotes. As positive and negative control we used β-glucan from *Candida albicans*^17^ and a non-stimulated RPMI medium condition, respectively. After 24 h of exposure with the first stimuli, the cells were challenged at day 6 with Toll-like receptor 4 (TLR4) (lipopolysaccharide [LPS]) or TLR2 (Pam_3_Cys) ligands as a secondary stimulus (Figure 1A). The exposure of monocytes to different concentrations (1, 10, 25, and 50 μg/mL) of *L. braziliensis* lysates led to increased production of TNF and interleukin-6 (IL-6) upon LPS restimulation 6 days later, measured as fold increase normalized to RPMI (Figure 1B). The concentrations of 50 and 25 μg/mL *L. braziliensis* lysates induced a 2-fold higher increase in TNF production than 1 μg/mL, and no significant differences were observed between lysates at 50 and 25 μg/mL. For IL-6, the exposure of monocytes to 50 μg/mL lysates led to a higher fold increase than with 1 μg/mL, and no differences were observed among the other doses of lysates (Figure 1B). Including β-glucan as a well-established inducer of TI allowed us to conclude that lysates of *L. braziliensis* induce TI based on the production of TNF and IL-6; therefore, we chose the dose of 25 μg/mL lysates to continue with in the *in vitro* experiments. Of note, the addition of Pam_3_Cys as a secondary stimulus resulted in similar effects on TNF release as observed with LPS (Figure 1C).

Next, we exposed primary monocytes to different ratios of live promastigotes of *L. braziliensis* per cell (MOI 50:1, 10:1, 5:1, or 2:1) to assess whether the live form of the parasite could exert lysate-like effects on monocytes. Upon LPS restimulation, we observed an increase in the fold induction of TNF and IL-6, indicating that the live forms of the parasites were also able to induce TI responses in monocytes *in vitro* (Figure S1A). We further characterized the ability of other *Leishmania* species to induce TI by exposing monocytes to lysates of *L. guyanensis* and *L. amazonensis*. Similar to *L. braziliensis*, both *L. guyanensis* and *L. amazonensis* enhanced the fold induction of TNF and IL-6 when the cells were restimulated with LPS. Interestingly, when the production of the anti-inflammatory cytokine IL-10 was assessed, a tendency to increased IL-10 production was observed in monocytes exposed to *L. amazonensis* in comparison to *L. braziliensis*-exposed monocytes (Figure S1B). The presence of lipophosphoglycan (LPG) in the *L. braziliensis* extracts was established by 1D ^1^H NMR analysis at 60°C (Figure S1C). We observed two anomeric proton resonances at 5.47 and 4.49 ppm and lipid resonances between 0.5 and 1.5 ppm. Based on 2D correlation spectroscopy analysis and comparison with the structural analysis of an LPG from *L. donovani*^24^ and *L. major*,^25^ these anomeric ^1^H resonances were assigned to αDManp and βDGalp repeat units, respectively, in the −6Galβ1-4Manα1-PO_4_- structural fragment of the LPG fraction. Based on these data, we conclude that *L. braziliensis* contains an LPG-like molecule. Other carbohydrate repeat units were not observed in the NMR spectrum. The addition of different concentrations of LPG to monocytes triggered increased TNF production upon LPS restimulation, suggesting the importance of this glycoconjugate in the induction of TI by *Leishmania* (Figure S1D).

To assess which receptors were involved in *L. braziliensis*-induced TI in human monocytes, we used antibodies and small molecules as inhibitors of pattern recognition receptors (PRRs) or their downstream signaling mediators. Monocytes were incubated in the presence of different inhibitors and controls 1 h prior the addition of *L. braziliensis* or β-glucan. Thereafter, the procedures of the *in vitro* protocol of training were followed as described previously (Figure 1A). To study whether endocytosis and complement receptor 3 (CR3)-mediated phagocytosis are involved in the induction of TI by *L. braziliensis*, we pre-incubated monocytes with cytochalasin B and a neutralizing antibody for CR3. The TI phenotype was assessed by the ability of trained monocytes to release TNF upon LPS restimulation. The blockade of the uptake of *L. braziliensis* lysates by cytochalasin B and the anti-CR3 antibody led to a significant decrease in the fold induction of TNF after LPS restimulation in comparison to the vehicle and isotype control, respectively (Figure 1D). We further investigated the role of TLRs, nucleotide oligomerization domain 1 (NOD1) and NOD2, and C-type lectin receptors in the induction of TI by *L. braziliensis*. The blockade of TLR2 and TLR4 using a neutralizing antibody (anti-TLR2) and the natural TLR4 antagonist *Bartonella quintana* LPS^26^ caused a significant reduction in the fold induction of TNF upon training with *L. braziliensis* (Figure 1E). The cytosolic NOD1/2 receptors are also important for *L. braziliensis*-induced TI, as the inhibition of their downstream signaling molecule RIP2 kinase by ponatinib resulted in a significant reduction of TNF production in comparison to the vehicle (Figure 1E). The blockade of several C-type lectin receptors with specific antibodies including dectin-2, mincle mannose receptor, and DC-SIGN did not result in changes in TNF production upon training with *L. braziliensis* (Figure S1E). The involvement of TLR4, NOD1/2, and dectin-1 receptors were also shown to be important for the induction of IL-6 by *L. braziliensis*-trained macrophages, as the blockade of these receptors in the same manner as mentioned above diminished the IL-6 production (Figure S1F). Similar to β-glucan (Figure S1G), the exposure of monocytes to neutralizing antibodies against dectin-1 or to Raf inhibitor led to a significant decrease in the fold induction of TNF in *L. braziliensis*-trained macrophages compared to the isotype control and vehicle conditions. The inhibition of spleen tyrosine kinase (Syk) signaling did not result in differences in the fold induction of TNF in trained macrophages (Figure 1F). Collectively, the engagement of multiple immune receptors is required for the increased TNF and IL-6 production induced upon training of monocytes with *L. braziliensis*.

### *L. braziliensis* modulates the transcriptional response of human monocytes

Next, we performed transcriptional characterization of *L. braziliensis* lysate-exposed monocytes across different time points of the *in vitro* protocol using RNA sequencing (RNA-seq). The time points constitute the basal expression levels at day 0 prior to the addition of the first stimulus and the initial exposure to the first stimulus at day 1, followed by culture media removal and differentiation to macrophages at day 6 in the presence or absence of LPS for 4 h (Figure 1A;^13^). More than 2.505 genes were differentially expressed (fold change >2.5, adjusted p value <0.05) in our model between either treatments or time points. Over the time course, the major changes in gene expression patterns were already seen by day 1 (principal component [PC] 1, 50.2% of the variance), which was most pronounced between RPMI and *L. braziliensis* lysate-treated monocytes, and by day 0 prior to stimulation (PC2, 21% of the variance) (Figure 1G). Of note, when merging the RNA-seq dataset from a previous study in which β-glucan and RPMI conditions were used with the samples of our current study, a similar transcriptional pattern was observed for β-glucan- and *L. braziliensis*-exposed monocytes where most differences were seen after 24 h of stimulation (Figure S2A). Among the genes that changed in expression after 24 h, 137 genes, mostly immune genes, were transiently induced by *L. braziliensis*. Moreover, another set of 416 genes, mostly metabolic genes and genes associated with cell differentiation, changed in expression gradually over time during the transition from monocytes to macrophages in both RPMI-exposed and *L. braziliensis*-treated cells. Of note, the differentiation process was faster in *L. braziliensis* than in RPMI-exposed cells (Figure 1H). Chemokine genes such as *IL8*, *CXCL5*, and *CCL2* were examples of genes that were transiently upregulated in *Leishmania*-exposed monocytes after 24 h and rapidly decreased in expression at day 6. Genes associated with cell metabolism including *succinate dehydrogenase complex subunit C* (*SDHC*), *CD36*, and *lipoprotein lipase* (*LPL*) had an increased expression in *Leishmania*-treated cells after 24 h and remained elevated on day 6. However, the expression levels of these genes were similar to the RPMI control condition at day 6. The genes associated with cell differentiation, *CD68*, *LAMP1*, and *CD276*, gradually increased in expression over time and remained higher in *Leishmania*-exposed macrophages at day 6 in comparison to the untreated RPMI condition (Figure 1I). We studied the overlap between the transcriptional response induced after 24 h of exposure with *L. braziliensis* and β-glucan using the dataset from Novakovic et al.^13^ and observed that 15.5% (162) of the genes were commonly induced by both stimuli, whereas 37.1% (391) and 47.5% (501) of the genes were specific for *L. braziliensis* and β-glucan, respectively (Figure S2B). The major ontologies of *L. braziliensis*-induced genes (381 genes at day 1) were metabolic pathways and peroxisome proliferator-activated receptor signaling (Figure 1J). The ontologies of genes downregulated by *L. braziliensis* (350 genes at day 1) were cytokine signaling, antigen processing and presentation, and circadian clock (Figure 1K).

Given that only few differences were observed in the overall transcriptional landscape of *L. braziliensis*-stimulated macrophages at day 6 in comparison to the control (Figure 1H), we sought to interrogate the gene responsiveness of RPMI and *L. braziliensis*-treated cells upon LPS restimulation for 4 h. Based on the mean and individual expression profile, most genes remained equal in expression when comparing *L. braziliensis*- and RPMI-treated macrophages upon LPS restimulation. However, some genes had higher (trained) and lower (tolerized) expression in response to LPS stimulation (Figure S2C). *IL12B*, *NFKB1*, and *IL1A* were examples of genes classified as trained, equal, and tolerized, respectively in *L. braziliensis*-trained macrophages after LPS stimulation (Figure S2D).

We used motif analysis to gain insights into which pathways and transcription factors were involved in the upregulation of transient, metabolic, and cell differentiation associated genes upon *L. braziliensis* exposure after 24 h. Interestingly, the strongest predicted motifs include basic-helix-loop-helix family members (BHLHE) 40/41 and clock circadian regulator CLOCK, which were associated with the transient upregulated subset of genes (Figure S2E). *L. braziliensis*-exposed cells induced the expression of *BHLHE41* (Figure S2F), and the accumulation of this transcription factor has been associated with increased expression of cytokines and proliferation genes in macrophages.^27^

### TI induced by *L. braziliensis* is mediated by JNK pathway, changes in metabolism, and chromatin accessibility

**Figure 2.**
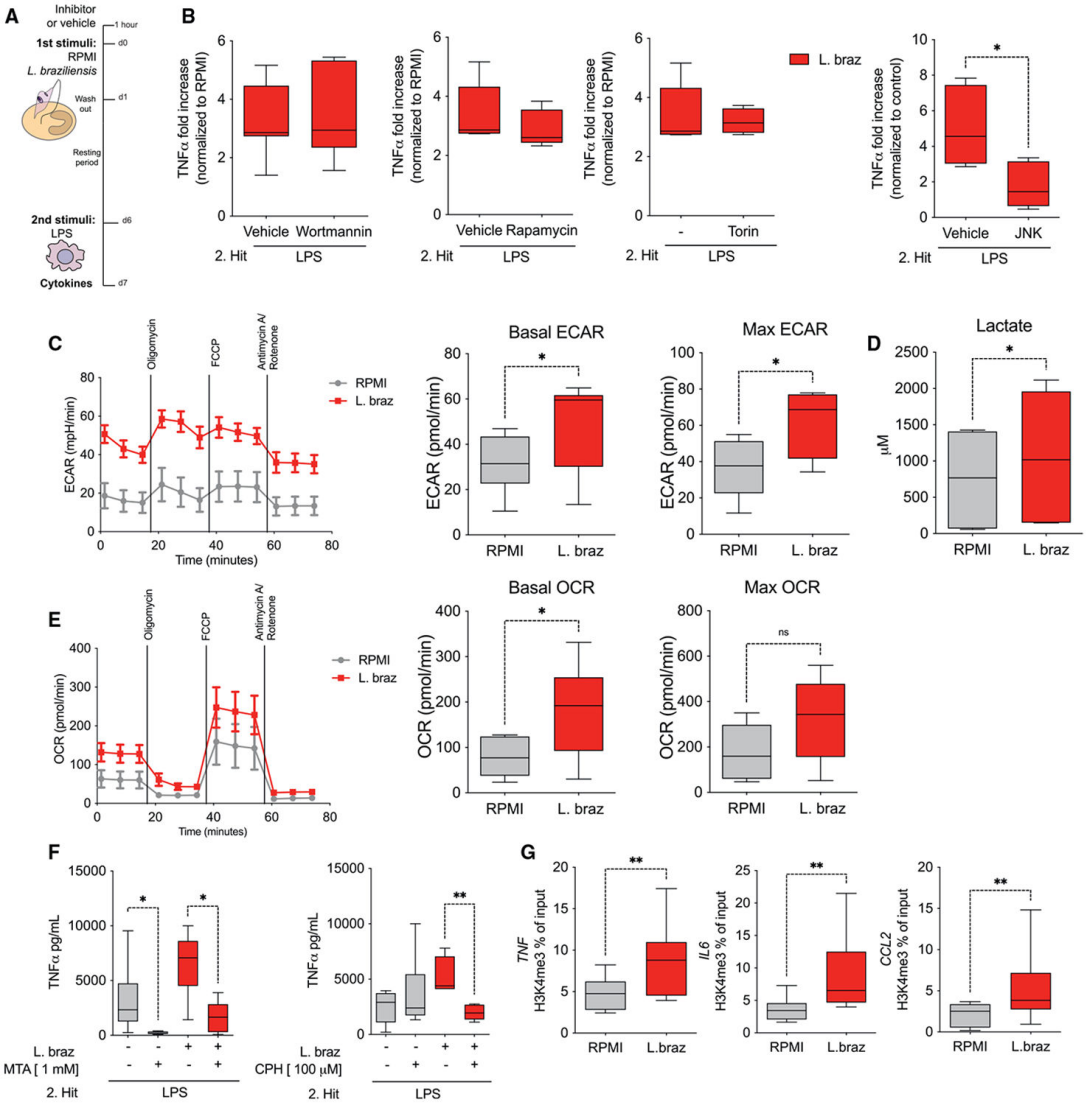
*L. braziliensis* training relies on JNK activation, metabolism, and H3K4me3 deposition (A and B) (A) Experimental setup. TNF production after LPS (10 ng/mL) restimulation at day 7 of *L. braziliensis*-trained (25 μg/mL) macrophages ± (B) the PI3K/AKT, mTOR inhibitors wortmannin and rapamycin/torin, respectively, and the JNK inhibitor SP600125. As controls, RPMI (−) or RPMI + DMSO (vehicle) were used as indicated. (C) Basal and maximum extracellular acidification rates (ECAR) of RPMI- and *L. braziliensis*-trained macrophages at day 6, measured by Seahorse. (D) Lactate production assessed in the supernatant of trained macrophages at day 6 by fluorometric assay. (E) Basal and maximum oxygen consumption rate (OCR) of RPMI- and *L. braziliensis*-trained macrophages at day 6, measured by Seahorse. (F) TNF production after LPS restimulation at day 7 of RPMI-exposed and *L. braziliensis*-trained (25 μg/mL) macrophages ± the methyltransferase inhibitors MTA and CPH. (G) Levels of H3K4me3 at *TNF*, *IL6*, and *CCL2* promoters of RPMI- and *L. braziliensis*-exposed macrophages assessed by chromatin immunoprecipitation-qPCR. n = 6 independent donors. Cytokine measurements are presented as fold increase normalized to RPMI (non-trained cells). Data are shown in box-and-whiskers (minimum-to-maximum) plots from two independent experiments (*p < 0.05; **p < 0.01 by Wilcoxon test; ns, p > 0.05). See also Figure S3.

To understand the mechanisms involved in the transcriptional response and increased cytokine release upon *L. braziliensis* exposure, we investigated the role of phosphatidylinositol 3-kinase (PI3K/AKT), mammalian target of rapamycin (mTOR), and c-Jun N-terminal kinase (JNK) pathways. We assessed LPS-induced TNF levels upon training of monocytes with *L. braziliensis* or RPMI in the presence or absence of specific inhibitors for the aforementioned pathways (Figure 2A). The PI3K/AKT and mTOR inhibition with wortmannin, rapamycin, and torin had no effect on the induction of TNF. However, the treatment of monocytes with a JNK inhibitor significantly decreased the fold induction of TNF, confirming the involvement of this pathway in the induction of TI by *L. braziliensis* (Figure 2B).

We next investigated the glucose metabolism and oxidative phosphorylation (OXPHOS) in monocytes trained with lysates of *L. braziliensis* by assessing basal and maximum extracellular acidification rate (ECAR), oxygen consumption rate (OCR), and the accumulation of lactate. The basal and maximum ECAR was higher in *L. braziliensis*-treated macrophages than in RPMI-treated cells, whereas for the OCR, significant differences were observed for basal rates only (Figures 2C and 2E). Moreover, an increase in lactate levels was observed in the supernatants of *L. braziliensis*-trained macrophages (Figure 2D). In line with previous reports,^28^ the addition of β-glucan, used as positive control in our study, also led to increased ECAR, lactate, and OXPHOS (Figures S3A-S3C). Of note, the inhibition of glycolysis and OXPHOS by deoxyglucose and oligomycin, respectively, abolished the increase in TNF production by *L. braziliensis*-trained macrophages (Figure S3D). Collectively, our results demonstrate the importance of glycolysis and OXPHOS in the induction of the trained phenotype induced by *L. braziliensis*.

To assess whether training of monocytes induced by *L. braziliensis* was dependent on changes in chromatin accessibility, we pre-incubated monocytes with methylthioadenosine (MTA, a pan-methyltransferase inhibitor) for 24 h prior the addition of *Leishmania* during the *in vitro* training protocol (Figure 2A). The presence of MTA abrogated the production of TNF by macrophages exposed to *L. braziliensis* and restimulated with LPS. Moreover, and based on a previous study identifying Set7 lysine methyltransferase (SET7) as an important regulator of TI induced by β-glucan,^28^ we tested the role of Set7 in *L. braziliensis*-induced TI by incubating monocytes with cyproheptadine (CPH) for 24 h. After training and restimulation with LPS, the levels of TNF were significantly decreased in the CPH-treated macrophages in comparison to the vehicle control (Figure 2F). Next, we assessed the enrichment of transcriptionally permissive histone modification (H3K4me3) at the proximal promoter regions of genes that have been linked to the increased responsiveness of *Leishmania*-trained monocytes including *TNF*, *IL6*, and *CCL2*. An increased deposition of H3K4me3 was observed on the promoters of *TNF*, *IL6*, and *CCL2* of *L. braziliensis*-trained macrophages at day 6 in comparison to RPMI controls (Figure 2G). We extended our analysis to metalloproteinase genes that were previously associated with the TI phenotype induced by oxLDL.^11^ A significantly increased deposition of H3K4me3 was observed for *MMP2* in cells exposed to *L. braziliensis* in comparison to RPMI. Although the differences in H3K4me3 levels were not significant for *MMP9*, a tendency to being increased was observed in the *L. braziliensis* condition (Figure S3E). These data highlight the importance of chromatin modifications in modulating the production of mediators associated with the increased responsiveness of macrophages during TI induced by *L. braziliensis*.

### *L. braziliensis*-trained macrophages are permissive for the infection with live promastigotes

**Figure 3.**
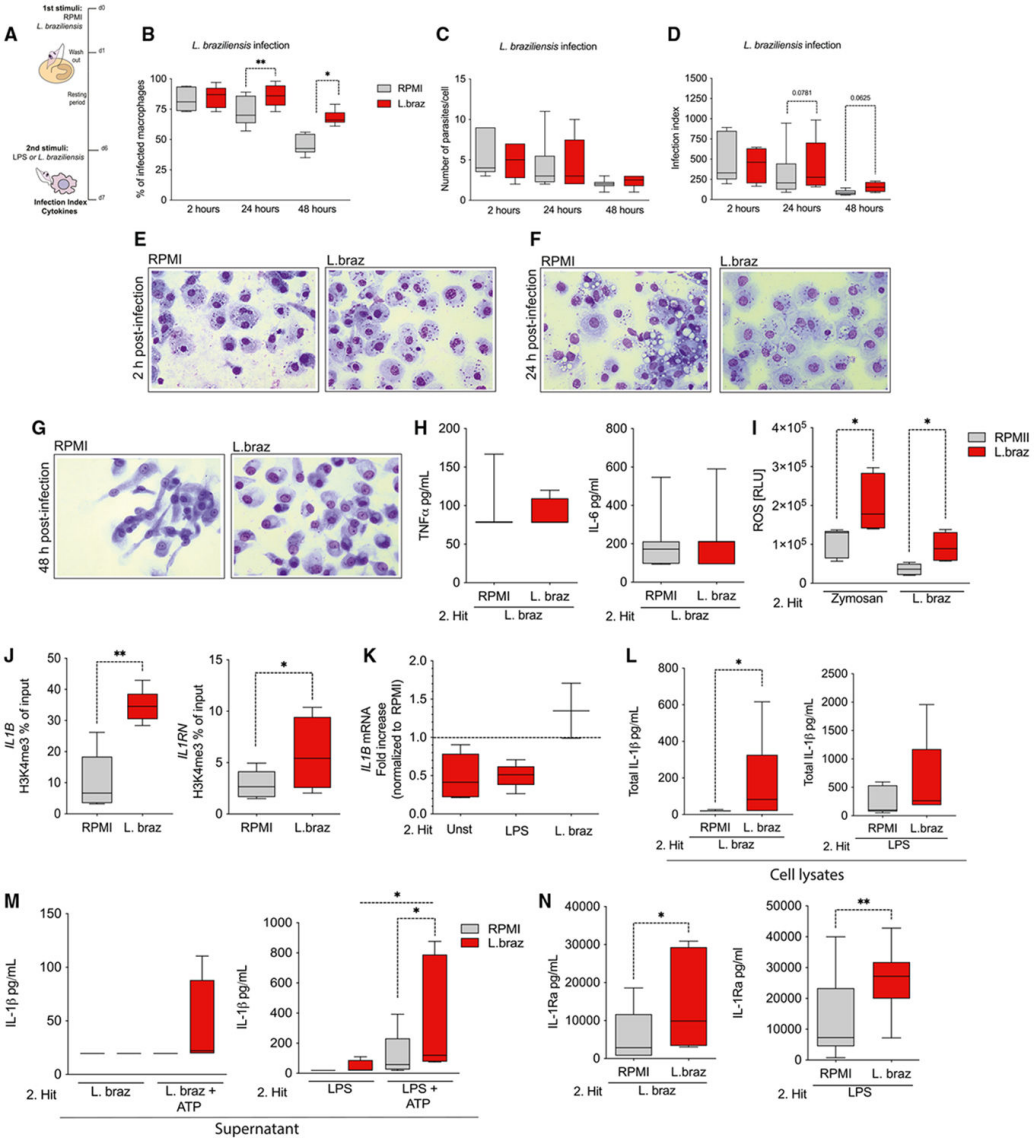
*L. braziliensis*-trained macrophages are permissive for intracellular growth of *Leishmania* amastigotes *in vitro* (A) Experimental setup. (B–D) (B) Percentages of infected macrophages, (C) number of parasites per infected cell, and (D) infection index were assessed in RPMI- and *L. braziliensis*-trained (25 μg/mL) macrophages on day 6, 2 h, 24 h, and 48 h post infection with *L. braziliensis* promastigotes (MOI 5) by Giemsa. (E–G) Representative figures of RPMI- and *L. braziliensis*-treated macrophages (E) 2 h, (F) 24 h, and (G) 48 h post infection captured by light microscope (20×). (H) TNF and IL-6 release from *L. braziliensis* lysates-trained macrophages (25 μg/mL) after exposure to *L. braziliensis* promastigotes (MOI 5) measured by ELISA at day 7, 24 h post infection. (I) Reactive oxygen species (ROS) represented as area under the curve of a total 1-h period of RPMI- and *L. braziliensis*-trained macrophages restimulated with zymosan (1 mg/mL, positive control) and *L. braziliensis* lysates (25 μg/mL). ROS production was assessed by luminol-enhanced chemiluminescence assay. (J) Levels of H3K4me3 at *IL1B* and *IL1RN* promoters of RPMI- and *L. braziliensis*-exposed macrophages assessed by ChiP-qPCR. (K and L) (K) *IL1B* mRNA expression and (L) total intracellular IL-1β production measured in cell lysates of *L. braziliensis*-trained (25 μg/mL) macrophages on day 6 after *L. braziliensis* lysates (25 μg/mL) and LPS (10 ng/mL) restimulation by qPCR and ELISA, respectively. (M) IL-1β secretion in the supernatant of RPMI- and *L. braziliensis*-trained macrophages after restimulation of LPS (10 ng/mL) and *L. braziliensis* lysates (25 μg/mL) for 23 h followed by an additional 1-h incubation with ATP (1.25 mM). (N) IL-1Ra secretion in the supernatant of RPMI- and *L. braziliensis*-trained macrophages after LPS (10 ng/mL) and *L. braziliensis* restimulation. Both IL-1β and IL-1Ra were assessed in the supernatants using ELISA. n = 6 independent donors. *IL1B* mRNA are presented as fold increase normalized to RPMI (non-trained cells). Data are shown in box- and-whiskers (minimum-to-maximum) plots from two independent experiments (*p < 0.05; **p < 0.01 by Wilcoxon test; ns, p > 0.05).

So far, we have provided experimental evidence that *L. braziliensis* can trigger an increased responsiveness of macrophages upon exposure to an unrelated secondary stimulus. We investigated whether the macrophages that underwent training with *L. braziliensis* lysates were able to control the intracellular growth of live promastigote forms of the same species. We addressed this question by infecting RPMI- and *L. braziliensis*-exposed macrophages with live promastigote forms of *L. braziliensis* at day 6, and thereafter the percentage of infected cells and number of parasites per cell were monitored after 2, 24, and 48 h of incubation (Figure 3A). In *Leishmania*-trained macrophages, a significant increase in the percentages of infected cells was observed after 24 h and 48 h of infection in comparison to the RPMI-exposed cells (Figure 3B). No significant differences were observed in the number of parasites per infected cell between the conditions at the aforementioned time points (Figure 3C). Of note, there was a tendency to a higher infection index in *L. braziliensis*-trained macrophages after 24 h and 48 h (p = 0.0781 and 0.0625, respectively) infected with promastigote forms in comparison to the RPMI controls (Figure 3D). A representation of how the macrophages were displayed 2 h, 24 h, and 48 h post infection with live promastigote forms of *L. braziliensis* is shown in Figures 3E-3G. In ATL, the balance between production of pro- and anti-inflammatory cytokines is associated with parasite control and disease immunopathogenesis.^29^ Interestingly, after assessing the TNF and IL-6 production of RPMI- and *L. braziliensis*-exposed macrophages followed by live *Leishmania* re-exposure, no significant differences were observed between conditions (Figure 3H). In addition, *L. braziliensis*-trained macrophages led to an increased production of reactive oxygen species (ROS) upon restimulation with zymosan, used as positive control, and *Leishmania* in comparison to RPMI (Figure 3I). Next, we assessed the deposition of H3K4me3 at *IL1B* and *IL1RN* promoters in RPMI- and *L. braziliensis*-trained macrophages. We observed a significant increase on the promoters of both *IL1B* and *IL1RN* of *L. braziliensis*-trained macrophages (Figure 3J). The enrichment of H3K4me3 on the promoter of *IL1B* contributed to an enhanced *IL1B* mRNA expression in trained macrophages after live *Leishmania* restimulation as well increased production of total intracellular IL-1β. No significant difference in total intracellular IL-1β production was found in trained macrophages restimulated with LPS (Figures 3K and 3L). Importantly, IL-1β secretion only occurred when ATP was added in combination with both LPS and *Leishmania* as secondary stimuli for additional triggering of inflammasome activation. The addition of ATP contributed to an increased release of IL-1β in the supernatants of trained macrophages in comparison to RPMI-exposed macrophages (Figure 3M). Additionally, we observed increased production of IL-1Ra in macrophages trained with *L. braziliensis* in comparison to RPMI upon restimulation with both LPS and live *Leishmania* (Figure 3N). Collectively, we have shown that live *L. braziliensis*, when used as a secondary stimulus, elicits a different cellular response in terms of TNF and IL-6 induction. Moreover, *L. braziliensis*-trained macrophages have an increased production of intracellular IL-1β in which an additional signal is required to trigger its release. These mechanisms together may favor parasite survival in *L. braziliensis*-trained macrophages.

### TI induced by *L. braziliensis in vivo* promotes anti-tumor activities

**Figure 4.**
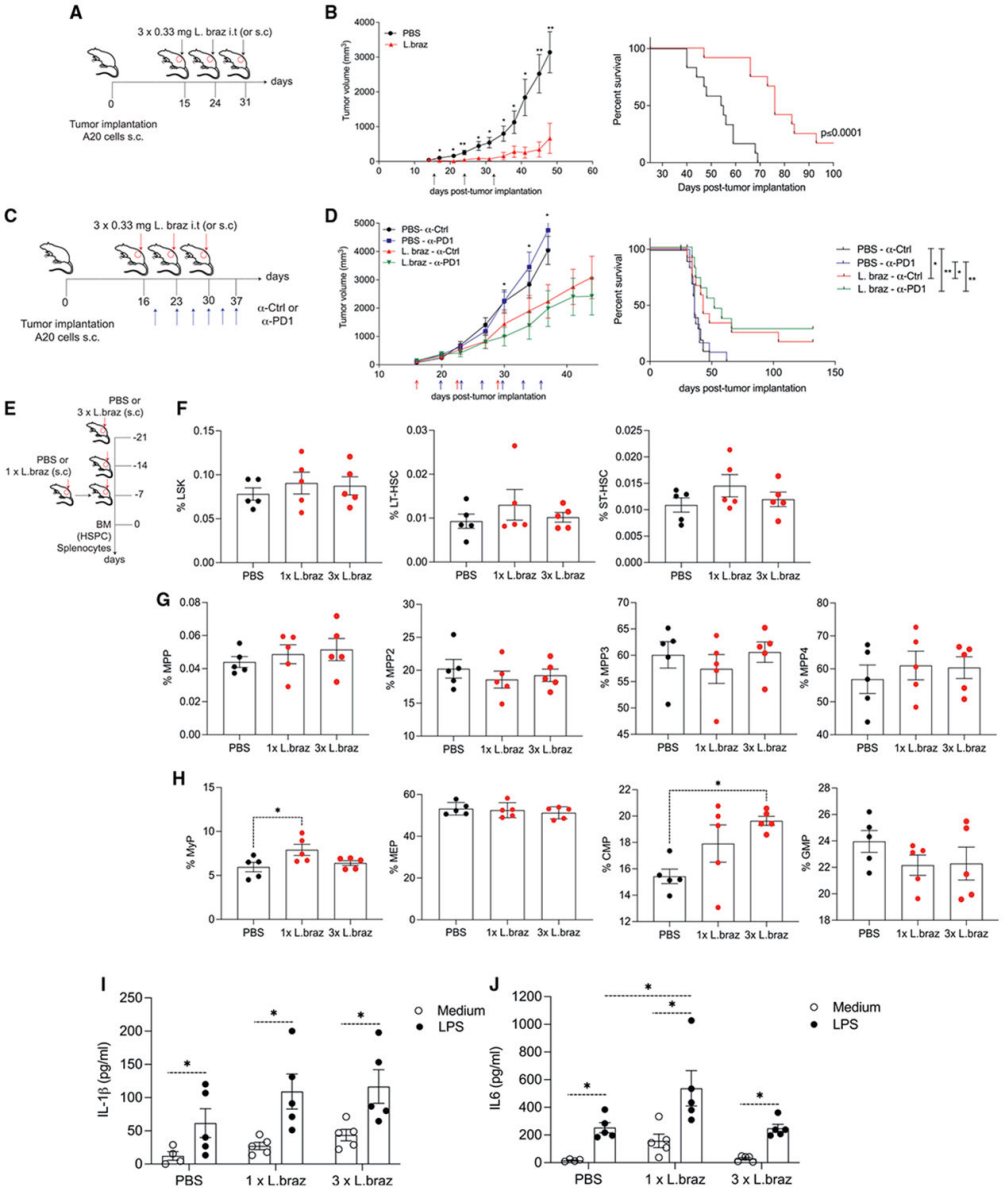
*L. braziliensis* injection delays tumor growth and prolonged survival of A20 non-Hodgkin-lymphoma-bearing mice (A) Experimental setup. (B) *In vivo* tumor growth and survival curves of BALB/c mice, which were inoculated with 1 × 10^6^ A20 tumor cells and submitted to a regimen consisted of three subcutaneous injections of PBS or *L. braziliensis* lysates (0.33 mg/mL each injection). Overall survival was followed up for 100 days (n = 12). Significance was calculated between the groups (*p < 0.05, ** p < 0.01 by t test and log rank, respectively). (C) Experimental setup of checkpoint inhibitor experiment. (D) *In vivo* tumor growth and survival curves of tumor-bearing BALB/c mice, which were randomized into one of the four treatment groups consisting of PBS and *L. braziliensis* in combination with anti-PD-1 (200 μg/mouse) and isotype control (200 μg/mouse) antibody regimens. The antibodies were administered intraperitoneally twice a week, starting after the administration of the first dose of PBS or *L. braziliensis* and lasted until day 37 post tumor implantation. Overall survival was followed up for 130 days (n = 12). Significance was calculated between all groups (*p < 0.05 by t test and log rank, respectively). (E) Experimental setup of *ex vivo* bone marrow phenotyping and splenocyte-derived cytokine production. Mice were injected with a single dose of *L. braziliensis* for 7 days or three doses of *L. braziliensis* or PBS every 7 days. Bone marrow cells were collected for *ex vivo* immunophenotyping analysis by flow cytometry. (F–H) Percentages of (F) LSKs, LT-HSCs, and ST-HSCs, (G) MMPs, MMP2, MMP3, and MMP4, and (H) MyPs, MEPs, CMPs, and GMPs in PBS- and *L. braziliensis*-treated mice (n = 5).. (I and J) Splenocytes (5 × 10^5^) of PBS- and *L. braziliensis*-treated mice submitted to both single- and triple-dose regimens were restimulated for 24 h with LPS (100 ng/mL). Production of (I) IL-1β and (J) IL-6 was assessed in the supernatant by ELISA (n = 5). Data in bar plots are shown as mean ± SD (*p < 0.05 by t test). See also Figures S4 and S5.

Having established *L. braziliensis* as an initial stimulus resulted in trained macrophages, we wanted to extend our findings to the potential anti-tumor responses of TI induced by *Leishmania* lysates *in vivo*. We used a therapeutic setting in which A20 (B cell lymphoma) tumor cells were injected subcutaneously in BALB/c mice (Figure 4A). We observed a significant tumor-growth inhibition exerted by the treatment of mice with *L. braziliensis* lysates in comparison to the PBS-treated group. Interestingly, the tumor volume of *L. braziliensis*-treated mice remained significantly smaller than that of the PBS group up to 40 days after treatment (Figure 4B). Of importance, after monitoring the tumor volume of each animal post tumor implantation, we noticed that most of the mice in the *L. braziliensis*-treated group had a substantial delay in tumor growth in comparison to animals receiving PBS (Figures S4A and S4B). In addition, animals treated with three doses of *L. braziliensis* lysates showed significantly prolonged survival compared to the PBS group (Figure 4B). To further explore whether the effects of *L. braziliensis* were applicable to other tumor models, we tested the effects of the lysates in the experimental model of melanoma. *L. braziliensis* lysates also exerted anti-tumor activities in the melanoma model, demonstrated by significant tumor-growth inhibition and prolonged survival in the animals treated with the lysates in comparison to the PBS group (Figures S4F and S4G).

We next performed experiments to compare anti-tumoral effects of immunotherapy with checkpoint inhibitors alone versus a combination with our TI-inducing *L. braziliensis* therapy. To explore this, we first performed an experiment to confirm the expression of programmed cell death-ligand 1 (PD-L1) in the A20 cell line (Figure S4C). Next, after 2 weeks of tumor implantation using A20 cells, mice were randomized and allocated to four treatment groups in which PBS and *L. braziliensis* was combined with anti-programmed cell death-1 (PD-1) and isotype control antibody regimens (Figure 4C). In the PBS-treated group, the anti-PD-1 therapy alone did not affect tumor growth in comparison to the PBS-treated isotype control group. Combining *L. braziliensis* with anti-PD-1 or isotype control significantly lowered tumor-growth rate compared to the PBS-treated groups with anti-PD-1 or isotype control regimens. We did not observe significant differences in tumor growth between *L. braziliensis*-treated groups combined with anti-PD-1 or isotype control (Figure 4D). As seen in the first experiments, we demonstrated that the treatment of tumor-bearing mice with *L. braziliensis* improved the survival of the animals to up to 100 days in comparison to the PBS groups, a process that was observed for both anti-PD-1 and isotype control *L. braziliensis*-treated groups (Figure 4D). Interestingly, after assessing the cytotoxic activity of immune cells against A20 cells in splenocytes taken either from PBS- or *L. braziliensis*-treated mice that survived the tumor and non-tumor exposed control mice (naive), we observed a significant increase in cytotoxic activity of splenocytes derived from *L. braziliensis*-treated mice when a 100:1 effector/target ratio was used (Figure S4D). Moreover, these increased cytotoxic effects were seen in splenocytes derived from *L. braziliensis*-treated mice in which the treatment was combined with anti-PD-1 and isotype control antibodies (Figure S4E).

So far, our results showed a potent ability of *L. braziliensis* lysates to inhibit tumor growth *in vivo*. We hypothesized that these anti-tumor effects were exerted through the amplification of myelopoiesis at bone marrow level, which is an intrinsic mechanism associated with the TI phenotype induced by β-glucan.^30^ Using a different animal group, we performed three additional experiments: (1) mice were injected with a single dose of *L. braziliensis*; (2) mice were injected with three doses of *L. braziliensis* or PBS every 7 days; and (3) functional responses of bone marrow cells were studied through the assessment of cytokine production upon LPS restimulation *ex vivo*. In both experiments, bone marrow cells were collected for *ex vivo* immunophenotyping analysis (Figures 4E and S5A). The subcutaneous injection of *L. braziliensis* lysates did not induce major changes in either the absolute numbers and percentages of total hematopoietic bone marrow progenitor cells (LSKs, Lin^−^cKit^+^Sca1^+^) or in the long- and short-term hematopoietic stem cells (LT-HSCs, CD48^−^CD150^+^LSK and ST-HSCs, CD48^−^CD150^+^LSK, respectively), as compared to PBS-treated mice (Figures 4F and S5B). Moreover, no differences were observed after the analysis was extended to the percentages and numbers of multipotent progenitors (MMPs, CD48^+^CD150^−^LSK) as well as of MMP2 (Flt3^−^CD48^+^CD150^+^LSK), MMP3 (Flt3^−^CD48^+^CD150^−^LSK), and MMP4 (Flt3^+^CD48^+^CD150^−^LSK) subsets among the PBS- and *L. braziliensis*-injected groups (Figures 4G and S5C). Seven days after one injection of *L. braziliensis*, an increase in the percentages of myeloid progenitors (MyPs, Lin^−^cKit^+^Sca1^−^) was observed in comparison to PBS-injected control mice. No differences were observed for the MyP absolute numbers (Figure S5C). Furthermore, the administration of three doses of *Leishmania* lysates led to increased proportions of common myeloid progenitors (CMPs, Lin^−^cKit^+^Sca1^−^CD16/CD32^−^CD34^+^) compared to control mice. The proportions of granulocyte-macrophage progenitors (GMPs, Lin^−^cKit^+^Sca1^−^CD16/CD32^+^CD34^+^) were not affected by the injection of one or three doses of *Leishmania* lysates (Figure 4H). No differences were observed in the production of TNF, IL-6, and IL-1β by bone marrow cells isolated from A20 lymphoma-bearing mice treated with PBS and *L. braziliensis* after LPS restimulation *ex vivo* (Figure S6H).

After investigating the effects of *L. braziliensis* lysates on bone marrow hematopoietic stem and progenitor cell (HSPC) expansion and composition, we assessed “peripheral” TI by evaluating the *ex vivo* response of splenocytes to LPS restimulation. IL-1β production by LPS-exposed splenocytes was greater than in splenocytes exposed to control medium in all three groups of mice. However, the production of IL-1β in PBS-treated versus *L. braziliensis*-treated groups did not differ (Figure 4I). Interestingly, the addition of LPS triggered higher IL-6 production in splenocytes from mice exposed to a single dose of *L. braziliensis* compared to the PBS-treated group. Similarly to IL-1β, the IL-6 concentrations were higher in LPS-exposed splenocytes than in control medium for all groups (Figure 4J). Subcutaneous injection of various doses of *L. braziliensis* mildly affects HSPC composition. Functionally, these cells do not exhibit enhanced cytokine production upon restimulation. Instead, *L. braziliensis* lysates induced peripheral TI by modulating splenocyte cytokine and cytotoxic responses.

### *L. braziliensis* induces the infiltration of myeloid and CD8^+^ T cells into the tumor

**Figure 5.**
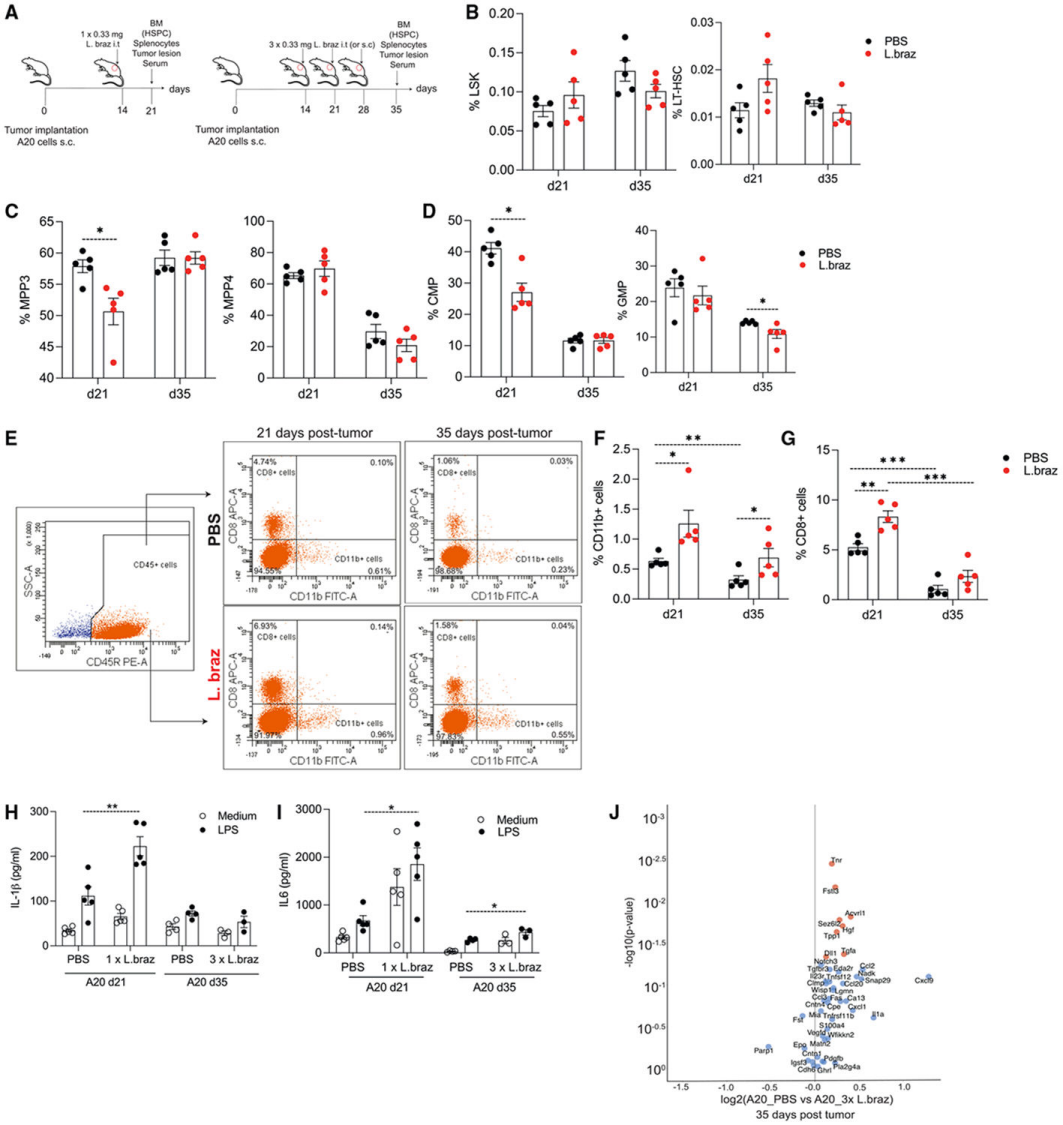
*L. braziliensis* injection promotes the infiltration of immune cells into the tumor (A) Experimental setup. *Ex vivo* bone marrow phenotyping of tumor-bearing PBS- and *L. braziliensis*-treated (0.33 mg/mL) mice submitted to both single-dose (day 21) and triple-dose (day 35) regimens injected subcutaneously. (B–D) Percentages of (B) LSKs and LT-HSCs, (C) MMP3 and MMP4, and (D) CMP and GMP were assessed by flow cytometry (n = 5). (E) Representative flow-cytometry plots. (F and G) Percentages of (F) CD45^+^CD11b^+^ and (G) CD45^+^CD8^+^ cells in the tumor lesion were assessed by flow cytometry (n = 5). 5 × 10^5^ splenocytes of were restimulated for 24 h with LPS (100 ng/mL). (H and I) Production of (H) IL-1β and (I) IL-6 was assessed in the supernatant by ELISA (n = 5). (J) Proteins measured in the serum by Olink in both tumor-bearing PBS- and *L. braziliensis*-treated mice 35 days after tumor implantation. Volcano plot showing the differentially expressed proteins between the two groups (log_2_ values; significant proteins are marked in red; false discovery rate adjusted *p < 0.05). Data in bar plots are shown as mean ± SD (*p < 0.05, ** p < 0.01 by t test). See also Figure S6.

After evaluating the effect of multiple doses of *L. braziliensis* lysates in the absence of the tumor, we continued our analysis to evaluate the central versus peripheral induction of TI 21 and 35 days after tumor implantation. We assessed the HSPC composition, the infiltration of CD11b^+^ and CD8^+^ cells into the tumor, the *ex vivo* production of cytokines by splenocytes, and the systemic release of inflammatory mediators in the serum of both PBS- and *L. braziliensis*-treated mice (Figure 5A).

Assessment of the bone marrow composition in mice exposed to one or three doses of *L. braziliensis* at days 21 and 35 did not show differences in the absolute numbers and percentages of LSKs, LT- and ST-HSCs, and MMPs in comparison to the PBS group (Figures 5B and S6B-S6E). In addition, there was a decrease in the percentages of the myeloid-biased MMP3 cells in *L. braziliensis*-treated mice21 days post tumor implantation, in comparison to the PBS-injected group. The percentages of lymphoid-based MMP4 and MMP2 cells were the same in both groups and at both time points post tumor implantation (Figures 5C and S6E). Analysis of MyP percentages did not show significant differences between the groups and time points (Figure S6F). Moreover, the CMP proportions were decreased at day 21 in *L. braziliensis*-treated mice, yet no significant differences between groups were observed at day 35. As for the GMPs, a significant decrease in the percentages of cells was observed in *L. braziliensis*-treated mice at day 35 post tumor implantation (Figure 5D). Collectively, our data indicate the presence of a negative regulation of the induction of myelopoiesis centrally at the bone marrow of *L. braziliensis*-treated mice after tumor implantation.

Next, we studied the influx of mature myeloid and lymphoid cells to the periphery, more specifically at the tumor environment, by assessing the presence of total CD45^+^, CD11b^+^, and CD8^+^ cells after 21 and 35 days of tumor establishment (Figure 5E). We observed an increase in the influx of CD11b^+^ into the tumor in *L. braziliensis*-treated mice at days 21 and 35 in comparison to the PBS group. At day 21, the proportion of CD11b^+^ cells in the tumor of PBS-treated mice were higher than at day 35 (Figure 5F). The injection of *L. braziliensis* significantly increased the influx of CD8^+^ cells at day 21 in comparison to the PBS group. Moreover, the proportions of CD8^+^ in the tumor decreased by day 35 in both PBS- and *L. braziliensis*-treated groups, demonstrating that this influx is transient (Figure 5G). Thereafter, we performed an independent experiment aiming to further characterize the phenotype of CD11b^+^ cells found to be increased in the tumor environment. Our results indicated a trend toward increased CD11b^+^F4/80^+^ macrophages in *L. braziliensis*-treated mice in comparison to the control group, whereas no differences were seen for CD11b^+^Ly6G^+^ cells between the groups (Figures S6G and S6I).

To investigate whether the peripheral cells displayed an increased responsiveness upon restimulation, we measured the *ex vivo* cytokine production of splenocytes upon LPS restimulation. The splenocytes of *L. braziliensis*-treated mice released higher amounts of IL-1β and IL-6 than the PBS control group at day 21 post tumor implantation. At day 35, no significant differences in the secretion of IL-1β was observed between the groups, whereas for IL-6, higher production was observed in the *L. braziliensis*-treated mice than in the PBS control group (Figures 5H and 5I). To assess the systemic effects of the injection of *L. braziliensis* lysates, we measured the presence of immune mediators in the serum of the animals at day 35 post tumor implantation. The results showed increased production of proteins associated with signal transduction (tenascin R), transforming growth factor β signaling (follistatin-like 3 and activin A receptor type II-like 1), regulation of cell chemotaxis, apoptosis, and inflammatory responses (hepatocyte growth factor) in *L. braziliensis*-treated mice 35 days after tumor establishment (Figure 5J). Taken together, our data suggest that not only do *L. braziliensis* lysates incite a trained phenotype in peripheral immune cells, but also the induced anti-tumor effects are exerted locally into the tumor in a transient manner, a mechanism which is likely relevant for future clinical applications in treating solid tumors.

## DISCUSSION

In this study, we describe for the first time the molecular mechanisms through which the parasite *L. braziliensis* induces TI, resulting in a robust innate host response after a secondary challenge. Training of monocytes with *L. braziliensis* lysates induced a significant increase in the production of cytokines, which was attributed to metabolic and epigenetic mechanisms. Although trained macrophages did not confer protection against reinfection with *Leishmania*, we demonstrated an anti-tumor response exerted by the parasites *in vivo*, which contributed to prolonged survival of mice bearing non-Hodgkin lymphoma.

We have shown in this study that stimulation with *L. braziliensis*-induced TI results in increased responsiveness of monocytes and macrophages upon binding of a secondary TLR ligand. Importantly, we observed that the nature of the secondary stimulus contributes to the effector responses exerted by trained macrophages, as we showed that cells were not able to respond with higher production of cytokines upon reinfection with *L. braziliensis* promastigotes. Therefore, even though *L. braziliensis* induces TI, different pathways are required for effective control of parasite growth. Similar to BCG, monocytes exposed to *L. braziliensis* increase their ROS production upon restimulation.^31,32^ The induction of TI by β-glucan was shown to be beneficial in controlling infections caused by *L. braziliensis* via the activation of the IL-32-IL-1β axis. Both IL-32 and IL-1β are important for the induction of microbicidal molecules against *Leishmania*.^32,33^ *L. braziliensis* used as a secondary stimulus on trained macrophages led to an increased production of intracellular IL-1β but did not increase the amounts of IL-1β released in the supernatant. IL-1β secretion by trained macrophages occurred only in the presence of ATP, which is required for inflammasome assembly leading to caspase-1 activation and cleavage of the intracellular pro-IL-1β.^34^ The presence of IL-1Ra in combination with the absence of IL-1β secretion, together with the low production of TNF, could lead to the impairment of macrophage-mediated responses, which are important for parasite killing. In ATL, proinflammatory cytokines act like a double-edged sword that may induce protection against secondary infections but at the same time may be implicated in severe tissue destruction typical of this infection.^35^

The transcriptional response of monocytes upon training with *L. braziliensis* showed dynamic expression of genes and pathways modulated over time. *L. braziliensis* amplified the expression of genes involved in chemokine signaling, cell metabolism, and cell differentiation. We showed that increased *CCL2* mRNA expression in *L. braziliensis*-trained cells was accompanied by an enhanced H3K4me3 deposition at the promoter region of *CCL2*. The presence of myeloid cells that are prompted to produce CCL2 has important implications for the induction of peripheral TI, as it can favor the influx of immune cells that exert effector responses.^36^ Moreover, changes in the induction of cellular metabolism are a hallmark of trained monocytes.^28^ In the present study, we showed that glucose metabolism is crucial for *L. braziliensis*-induced TI. To accommodate the increased energy requirements needed for enhanced immune responsiveness, *L. braziliensis*’ induction of TI is dependent on the use of glycolysis and oxidative phosphorylation. Moreover, the increased inflammatory responses observed in human monocytes upon training with *L. braziliensis* were shown to be associated with the enrichment of transcriptionally permissive H3K4me3 chromatin modification at the promoter sites of the immune genes *TNF*, *IL6*, *IL1B*, *IL1RA*, and *MMP2*. Thus, our data indicate that the enhanced responsiveness of trained monocytes by *L. braziliensis* is dependent on complementary transcriptional, metabolic, and epigenetic programs. Importantly, even though the phenotype of trained macrophages induced by *L. braziliensis* shares similarities or additive effects when compared to well-established inducers of TI such as β-glucan and BCG, we also identified unique features that are specific for an *L. braziliensis*-induced TI program (including specific genes and PRRs). Moreover, synergistic effects of these inducers in exerting beneficial effects are shown by studies of patients with diffuse cutaneous leishmaniasis who were treated with BCG in combination with promastigote antigens of *Leishmania* spp., allowing control of parasites and long periods without relapses.^37,38^

The beneficial effects of TI were demonstrated in two independent studies using a B16-F10 melanoma model *in vivo*, in which the treatment of melanoma-bearing mice with either β-glucan or MTP-HDL (muramyl tripeptide-high-density lipoprotein) inhibited tumor growth.^39,40^ Moreover, TI induced by MTP-HDL was shown to potentiate the checkpoint blockage therapy using anti-PD-1.^40^ Likewise, we demonstrate in the present study that *L. braziliensis* immunotherapy can delay tumor growth and improve the survival rate of non-Hodgkin-lymphoma-bearing mice. Moreover, *L. braziliensis*-treated mice exerted the same effects in inhibiting tumor growth when compared with *L. braziliensis* + anti-PD-1-treated mice. In accordance with the literature, anti-PD-1 therapy alone had no survival benefit in the PBS group while the combination of *L. braziliensis* and anti-PD-1 induced a better survival outcome. Unresponsiveness to PD-1/PD-L1 blockade therapies has been reported previously in advanced stages of lymphoma, a process that was overcome by local delivery of an armed anti-PD-L1 antibody with type I interferon-α to create feedforward responses.^41^

TI-mediated long-term effects on myeloid cells occur via the modulation of progenitor cells centrally in the bone marrow, and these cells can transfer their trained phenotype to circulating immune cells.^42^ In addition, circulating monocytes and tissue-resident macrophages can develop innate immune memory at the tissue level, a process known as peripheral induction of TI. In the melanoma model, anti-tumor activities were achieved upon administration of β-glucan and MTP-HDL intraperitoneally and intravenously, respectively.^39,40^ In both cases, the involvement of myeloid progenitors was shown to be associated with the induction of TI. We have assessed the anti-tumor responses of *L. braziliensis* using a subcutaneous model in which the parasite fragments were injected intratumorally. We showed that the injection of the parasite lysates subcutaneously had mild effects centrally at the level of bone marrow progenitors. Importantly, we suggest the presence of induction of peripheral TI, as splenocytes of *L. braziliensis*-treated mice were able to build a stronger inflammatory response upon restimulation with LPS, a process that was exerted in the absence of tumor and in non-Hodgkin-lymphoma-bearing mice. In addition to cytokine release, we showed that these cells have a greater ability to exert cytotoxicity to tumor cells. In fact, in a recent study, the authors reported that β-glucan-trained macrophages can be activated upon exposure to tumor cells, contributing to the enhancement of anti-tumor responses such as increased cytotoxicity against pancreatic tumor cells.^36^ Locally at the tumor site, the anti-tumor activities in our model are likely to be attributed to the influx of myeloid cells characterized by the accumulation of CD11b^+^ cells and CD8^+^ T cells.

In conclusion, we provide comprehensive evidence that *L. braziliensis* exposure of human monocytes induces TI. The identification of the metabolic pathways and epigenetic markers contributing to induction of TI by *L. braziliensis* improves our understanding of pathogenesis of leishmaniasis and identifies potential therapeutic strategies for other non-related infectious diseases and cancer. Importantly, we demonstrate a capability of *L. braziliensis* lysates to exert anti-tumor responses locally by engaging myeloid cells *in vivo*. These findings emphasize the potential of using *L. braziliensis* lysates to therapeutically target myeloid cells within the tumor environment through the induction of peripheral TI. Thus, our results open the possibility to extend the life of patients with therapy for non-responsive malignancies by using agents that induce TI.

### Limitations of the study

Further studies aiming to investigate the effects of different doses of *L. braziliensis* lysates, as well as exploring the impact of the route of administration in the induction of anti-tumor activities *in vivo*, are of interest. Additionally, these studies may further elucidate the mechanisms associated with the induction of TI by *Leishmania* lysates *in vivo*, whether it occurs centrally at the level of progenitor cells or locally in peripheral tissues.

## STAR★METHODS

### RESOURCE AVAILABILITY

#### Lead contact

Further information and requests for resources and reagents should be directed to and will be fulfilled by the lead contact, Leo Joosten (leo.joosten@radboudumc.nl). This study did not generate unique reagents. All data are available in the main text or the supplementary materials.

#### Materials availability

The materials generated in this study are available from the lead contact upon request.

#### Data and code availability

- RNA-sequencing data have been made available on the Gene Expression Omnibus (https://www.ncbi.nlm.nih.gov/geo), series number GSE211212, and are publicly available as of the date of publication.
- This paper does not report original code.
- Any additional information required to reanalyze the data reported in this paper is available from the lead contact upon request.

### EXPERIMENTAL MODEL AND STUDY PARTICIPANT DETAILS

#### Human subjects

The study was approved by the Ethics Committee of Radboud University Nijmegen, the Netherlands (no. 42561.091.12). Experiments were conducted according to the principles expressed in the Declaration of Helsinki. All blood donors (Sanquin Blood Bank, Nijmegen, The Netherlands) gave written informed consent before donating the blood.

#### Animals

*In vivo* experiments were performed in 6–8 weeks old, female BALB/c or C57Bl/6 mice, which were housed on 12:12 h light/dark cycles and given food and water *ad libitum*. The experiments with animals were approved by the University’s Ethical Committee for Animal Experimentation, Uruguay (Exp. no. 070153-000830-20, protocol title “Evaluación de la inmunidad innata entrenada en cancer”, no. 1026).

#### Tumor cell lines

The A20 cell line (American Type Culture Collection, Manassas, USA) was routinely grown and maintained as previously described.^43^ PD-L1 expression on A20 cells was performed using anti-PD-L1-biotin (clone MIH5), followed by streptavidin-APC (BD Bioscience). The B16F10 cell line (American Type Culture Collection, Manassas, USA) was routinely grown and maintained as suggested by the supplier.

#### Leishmania parasites

*L*. (*V*.) *braziliensis* (MHOM/BR/2003/IMG), *L*. (*V*.) *guyanensis* (MHOM/BR/2006/PLR6), both strains from *Leishbank* IPTSP/UFG (Goiás, Brazil), and *L. amazonensis* (IFLA/BR/67/PH8), a reference strain, were used. Promastigote forms were cultured in Grace’s insect medium (Gibco, Life Technologies, USA) supplemented with 20% of heat-inactivated fetal bovine serum (FBS, Gibco, Life Technologies, USA) and 100 U/mL of penicillin/streptomycin (Sigma-Aldrich) at 26°C. Stationary-phase parasites were obtained on the 6^th^ day of growth and washed three times with phosphate-buffered saline (PBS; 1000xg, 10 min 10°C). After, they were suspended in PBS and quantified by using hemocytometer after fixation with PBS/0.4% formaldehyde. Live parasites were used in macrophage infection experiments. In addition, parasite lysates were obtained by 5 freeze-thaw cycles of promastigotes in the presence of protease inhibitors (Protease inhibitor cocktail, Sigma-Aldrich) in liquid nitrogen followed by thawing in a water bath at 37°C. Protein quantification was performed by using Pierce BCA protein assay kit (ThermoFisher Scientific, USA). The extraction and purification of *L. braziliensis* lipophosphoglycan (LPG) were performed as described in.^44,45^ Briefly, stationary-phase parasites were obtained on 6^th^ day of growth and washed three times with PBS. The LPG extraction with solvent E (MilliQ/Ethanol/Diethylether/Pyridine/NH_4_OH 25%; 15:15:5:1:0.017) over multiple centrifugation steps. For purification, the extract was dried under N_2_ evaporation, resuspended in 0.1 M acetic acid (AcOH or C_2_H_4_O_2_)/0.1M sodium chloride (NaCl) in MilliQ over hydrophobic chromatography using a column of phenyl-Sepharose. The column was washed with 0.1 M acetic acid/0.1M NaCl, 0.1 Macetic acid and MiliQ, respectively. The purified LPG was eluted in solvent E and dried under N_2_ evaporation.

### METHOD DETAILS

#### NMR structural analysis of LPG derived from *L. braziliensis*

The crude LPG extract was passed through a Sephadex G-25 column and the molecule of interest was eluted in the void volume. The carbohydrate was identified and structurally characterized by NMR analysis as described by Lowman et al.^46^ In summary, ^1^H NMR spectra for the *Leishmania* fractions were collected on a Bruker Avance III 400 NMR spectrometer operating at 294 K (21°C) and 333 K (60°C) in 5-mm NMR tubes. LPG fractions (about 7 mg) were dissolved in about 600 μL D_2_O (Cambridge Isotope Laboratories, 99.8+% deuterated). Chemical shift referencing was accomplished relative to TMSP at 0.0 ppm. 1D NMR spectra were collected as follows: 5000 scans, 65,536 data points, 20.69 ppm sweep width centered at 6.175 ppm, 1 s pulse delay and processed using exponential apodization with 1.0 Hz line broadening. 2D COSY spectra were collected as 2048 by 128, processed as 1024 by 1024, 16 pre-scans, 256 scans, sweep width 10 ppm centered at 4.5 ppm, and relaxation delay 1.49 s using sine apodization in both dimensions. Data processing was accomplished using TopSpin (version 4.0.9) on the MacBook Pro running macOS Catalina (version 10.15.7).

#### Peripheral blood mononuclear cells (PBMC), monocyte training and treatments

Human peripheral blood mononuclear cells (PBMCs) were isolated by dilution of blood in pyrogen-free PBS and differential density centrifugation over Ficoll-Paque (GE healthcare, UK) as previously described.^47^ Percoll isolation of monocytes was performed as previously described.^48^ Briefly, 150–200 × 10^6^ PBMCs were layered on a hyper-osmotic Percoll solution (48.5% Percoll, 41.5% sterile water, 0.16 M NaCl) and centrifuged for 15 min at 580 g (4°C). The interphase layer was collected, and cells were washed with cold PBS. Cells were resuspended in RPMI 1640 culture medium (Roswell Park Memorial Institute medium; Invitrogen, USA) supplemented with 50 μg/mL gentamicin, 2 mM glutamax (Gibco, Life Technologies, USA), and 1 mM pyruvate (Gibco) and quantified. Adherent monocytes were trained as described previously (depicted in Figure 1).^49^ Briefly, 100.000 cells were incubated either with culture medium containing 10% pooled human serum, referred to as complete medium, as a negative control, 5 μg/mL of β-glucan (β-1,3-(D)-glucan (kindly provided by Professor David Williams, College of Medicine, Johnson City, USA), lysates of *L. braziliensis* (50 μg/mL, 25 μg/mL, 10 or 1 μg/mL), lysates of *L. guyanensis* (25 μg/mL), or lysates of *L. amazonensis* (25 μg/mL). After 24 h (37°C), cells were washed once with 200 μL of warm PBS and incubated for 5 days with one change of complete medium. On day 6, cells were restimulated with either 200 μL RPMI, 10 ng/mL of lipopolysaccharide (LPS) derived from *E. coli* serotype O55:B5 (LPS - Sigma), or 10 μg/mL of Pam_3_Cys (Sigma) for 24 h. To assess the ability of live forms of *L. braziliensis* in inducing TI, monocytes were also incubated with stationary-phase promastigotes (MOI 50:1, 10:1, 5:1, 2:1 parasite/cell) were also used for 24 h. For experiments of IL-1β secretion, on day 6, cells were restimulated with RPMI, 10 ng/mL of LPS, 50 μg/mL of *L. braziliensis* for 23 h followed by an additional 1 h incubation with 1.25 mM ATP (Sigma). Supernatants were collected and stored at −20°C.

In inhibition experiments, monocytes were pre-incubated with the inhibitors 1 h prior to adding the stimuli (lysates of *L. braziliensis*). The metabolic and epigenetic inhibitors used were 1 mM 2-deoxyglucose (2-DG, Sigma), 10 μM oligomycin (Sigma), 1 mM methyltioadenosine (MTA - Sigma), or 100 μM cyproheptadine (CHP - Sigma). For receptor blockade experiments, 10 μg/mL of antibodies anti-TLR2 (InvivoGen), dectin-1, dectin-2, mincle, mannose, complement receptor 3 (CR3), and DC-SIGN (R&D systems) with its respective isotype controls were used. *Bartonella quintana* LPS^50^ (5 μg/mL) was used for inhibiting TLR4. 1 μg cytochalasin B (Sigma) and 100 nM Ponatinib (Selleckchem) were used for inhibiting endocytosis and RIP2 kinase, respectively. Spleen tyrosine kinase and serine-threonine kinase Raf-1 were inhibited by 50 nM of R406 (EMD Millipore) and 1 μM of GW5074 (Raf-Sigma), respectively. The c-Jun N-terminal kinase pathway was blocked by 10 μM of SP600125 (JNK-InvivoGen). Phosphatidylinositol 3-kinase (PI3K/AKT) and mTOR were blocked by 100 nM wortmannin (InvivoGen), 10 nM rapamycin (LC laboratories), and 100 nM torin (InvivoGen), respectively. In all experiments, either RPMI or RPMI+DMSO (vehicle) were used as negative controls.

For experiments of oxygen consumption rate (OCR) and intracellular acidification rate (ECAR), 10 × 10^6^ cells were trained *in vitro* in petri dishes (Corning, NY, USA) in 10 mL medium for 24 h as described above. On day 6, cells were detached from the plate with versine (Gibco) and counted. The analyses were performed as described below.

#### Cytokine and lactate measurements

Cytokine production was determined in supernatants using commercial ELISA kits (R&D Systems) for human TNF, IL-6, IL-10, IL-1β, and IL-1Ra. Total intracellular IL-1β was measured in cell-lysates collected in Triton X-100 0.5% (Sigma). Lactate concentration was measured in supernatants of human macrophages using Lactate Fluorometric Assay kit (Biovision, CA, USA).

#### Reactive oxygen species (ROS) assessment

Monocytes were isolated and trained with lysates of *L. braziliensis* (25 μg/mL) as described above. On day 6, ROS were measured by luminol (5-amino-2,3, dihydro-1,4-phtalazinedione - Sigma). A total of 1 × 10^5^ cells were seeded in white-96 well assay plate (Corning) in a volume of 100 μL. Next, the cells were stimulated with 25 μg/mL of *L. braziliensis* or 1 mg/mL of serum-treated zymosan (Sigma), used as a control of ROS production. Immediately after adding the stimuli, 1 mM of luminol diluted in hanks balanced salt solution (HBSS - Gibco) containing 0.5% of bovine serum albumin (BSA- Sigma) was added to each well. Chemiluminescence was measured in BioTek Synergy HT reader. The reading was performed at 37°C for every minute for 1 h.

#### RNA sequencing and differential gene expression analysis

For obtaining the RNA, monocytes were isolated with magnetic activated cell sorting with MACS pan monocyte isolation (Miltenyi Biotec) and trained with *L. braziliensis* lysates (25 μg/mL) as described above. Cells were collected at the relevant time points and harvested in RLT buffer (Qiagen) before freezing at −80°C. RNA was isolated using RNeasy mini columns (Qiagen) according to the manufacturer’s instructions. RNA sequencing library preparation was performed using the Quantseq 3′ mRNA-Seq Library Prep Kit-FWD from Lexogen (Lexogen, 015.96, Vienna, Austria) according to the manufacturer’s instructions. Sequencing of the libraries was performed on a NextSeq 500 instrument (Illumina, San Diego, CA, USA). RNA-seq reads were mapped to the hg19 human reference genome using GSNAP.^51^ To infer gene expression levels, RNA-seq reads were aligned to the Ensembl v68 human transcriptome using Bowtie.^52^ Quantification of gene expression levels as RPKM was performed using MMSEQ.^53^ Differential gene expression analysis was performed with DESeq2 v1.22.0, with a fold change of >2.5 for genes with >5 RPKM considered differentially expressed. An average expression value was generated by combining the three donors for each condition across each time point. We then performed Gene Ontology analysis on a dynamic list of genes using a Gene Functional Classification Tool (DAVID).^54^ The DNA-binding motif analysis was performed as described by.^13^

#### Assessment of oxygen consumption and acidifications rates

Real-time analysis of ECAR and OCR on monocytes was performed using an XF-96 Extracellular Flux Analyzer (Seahorse Bioscience) as previously described.^55^ Monocytes were isolated and trained with lysates of *L. braziliensis* (25 μg/mL) as described above. On day 6, 200.000 macrophages/well were seeded in quintuplicate in XF-96 cell culture plates in the presence of XF Base Medium (unbuffered DMEM with 5.5 mM glucose and 2 mM L-glutamine, pH adjusted to 7.4). The metabolic rates of monocytes were analyzed in four consecutive measurements. After three basal measurements, three consecutive measurements were taken following the addition of 1.5 μM oligomycin, 1 μM carbonyl cyanide-4-(tri-fluoromethoxy) phenylhydrazone (FCCP), and 2 μM antimycin together with 1 μM rotenone to determine basal and maximum OCR and ECAR. All compounds used during the Seahorse runs were acquired from Sigma.

#### Chromatin immunoprecipitation (ChIP)

Monocytes were isolated and trained with lysates of *L. braziliensis* (25 μg/mL) as described above. On day 6, cells were harvested and fixed in 1% methanol-free formaldehyde. Fixed cell preparations were sonicated using Diagenode Bioruptor Pico sonicator and immunoprecipitated using antibodies against H3K4me3 (Diagenode, Seraing, Belgium). A MinElute PCR purification Kit (QIAGEN) was used for DNA isolation. qPCR analysis was performed using an SYBR Green method, and samples were analyzed by a comparative Ct method. Myoglobin was used as negative control and H2B as a positive control according to the manufacturer’s instructions. Primer sequences are described in.^11,20^

#### Evaluation of macrophage infection

Monocytes were trained with lysates of *L. braziliensis* as described above (25 μg/mL). 2 × 10^5^ cells were added to 24-well plates on 12 mm coverslips (Corning), adhered for 1 h at 37°C, and infected with stationary-phase promastigotes of *L. braziliensis* at 1.0 × 10^6^/well (MOI 5:1) in a final volume of 500 μL complete medium. Coverslips were collected after 2-, 24-, and 48-h incubation. The cells were fixed with methanol (Sigma) and stained by Giemsa (Merck Millipore). 300 cells were analyzed under a light microscope, and the percentage of infected cells and the mean number of intracellular parasites per infected cell (at least 50) were determined. Infection index = percentage of infected cells × mean number of parasites per infected cell.

#### *In vivo* L. *braziliensis*-induced trained immunity *Tumor inoculation and treatment regimen*

The A20 and B16F10 cell lines were grown in culture and harvested in log phase. Then cells were washed and resuspended to a final concentration of 5 × 10^6^ cells/mL and 2.5 × 10^6^ cells/mL in PBS for A20 and B16F10, respectively. Balb/C or C57Bl/6 mice were injected subcutaneously (s.c.) into the right flank with 1 × 10^6^ A20 cells in 0.2 mL of PBS or 2.5 × 10^5^ B16F10 cells in 0.1 mL of PBS, respectively. Animals were divided in 2 groups (n = 12 per group): control (PBS) and lysate of *L. braziliensis* (L. braz) groups. On day 15 post-tumor implantation (p.t.i.) for A20 lymphoma model or 10 p.t.i. for B16F10 melanoma model, mice received a subcutaneous injection of 0.33 mg of lysate of *L. braziliensis* or PBS as control (in 100 μL). Afterward, as tumors become palpable, mice received a weekly intratumoral (i.t.) injection of 0.33 mg of lysate of *L. braziliensis* or PBS for two weeks (days 24 and 31 p.t.i. for A20 model or 17 and 24 p.t.i. for melanoma model). For experiments involving checkpoint inhibition therapy, 200 mg anti-PD-1 (clone, RMP1-14, BioXcell) or isotype control was injected intraperitoneally twice a week. The treatment started after the first injection of PBS or *L. braziliensis* lysates injection and continued until day 37 p.t.i. Tumors were measured every other day with a microcaliper, and tumor volumes were calculated as length × width × depth × π/6.^56^ Euthanasia was carried out by cervical dislocation when tumors reached 4,000 mm^3^ or earlier if animals showed signs of distress.

#### Bone marrow phenotyping and flow cytometry

To study the effect of *L. braziliensis* lysates on hematopoietic progenitor cells mice received a single or three subsequent subcutaneous injections of 0.33 mg of lysate of *L. braziliensis* or PBS as control (in 100 μL) (n = 5 per group); mice were euthanized 7 days after the last injection (day 7 and 21). In another experiment, mice were injected with A20 cells as described above, and after 14 days, the animals received one or three i.t. injections of 0.33 mg of lysate of *L. braziliensis* or PBS; mice were euthanized 21 and 35 days p.t.i. (e.i. 7 days after the first and third dose, respectively). The bone marrow (BM) was flushed from the femur using a 5% PBS-FBS solution. BM surface phenotype analysis was performed as described by.^30^ Cell suspensions were incubated with several antibody cocktails including a lineage cocktail (Lin) containing anti-CD3e (clone 145-2C11), anti-CD11b (clone M1/70), anti-Gr1 (clone RB6-8C5), anti-B220 (clone RA3-6B2), anti-TER119 (clone TER-119), anti-Sca1 (clone E13–161.7), anti-cKit (clone 2B8), anti-CD135 (clone A2F10), anti-CD48 (clone HM48-1), anti-CD150 (clone TC15-12F12.2), anti-CD16/CD32 (clone 93), and anti-CD34 (clone RAM34), and a lineage cocktail (Lin) containing anti-CD3 (clone 17A2), anti-CD11b (clone M1/70), anti-Gr1 (clone RB6-8C5), anti-CD45R/B220 (clone RA3-6B2), and anti-TER119 (clone TER-119). All data were acquired on FACSCanto II (BD Bioscience) and data analysis was performed using Diva software (BD Bioscience). On another experiment, 1 × 10^5^ BM cells were stimulated with 10 ng of LPS from *E. coli* (Invivogen) for 24 h. Supernatant were collected and stored at −80°C until analyzed. Cytokine production was determined using commercial ELISA kits (R&D SystemsBiolegend) for mouse TNF, IL-1β and IL-6.

#### Tumor flow cytometry

To study the effect of *L. braziliensis* lysates on tumor infiltrating cells, A20-lymphoma bearing mice received a single or three subsequent subcutaneous injection of 0.33 mg of lysate of *L. braziliensis* or PBS as control (in 100 μL) (n = 5 per group) as described above. Mice were euthanized 7 or 21 days later, and tumors were removed and processed as previously described.^57^ Cell suspensions were incubated with anti-CD45 (clone 30-F11), anti-CD11b (clone M1/70), anti-CD8 (clone 53–6.7), anti-Ly6G (clone 1AS) and anti-F4/80 (clone QA17A29). All data were acquired on FACSCanto II (BD Bioscience) and data analysis was performed using Diva software (BD Bioscience).

#### Ex vivo *cytokine production and cytotoxic capacity of splenocytes*

To study the effect of *L. braziliensis* lysates on induction of trained immunity on splenocytes, mice received a single or three subsequent subcutaneous injection of 0.33 mg of lysate of *L. braziliensis* or PBS as control (in 100 μL) (n = 5 per group); mice were euthanized 7 days after the last injection (day 7 and 21). In other experiment, mice were injected with A20 cells as described above and after 14 days, the animals received one or three i.t. injection of 0.33 mg of lysate of *L. braziliensis* or PBS; mice were euthanized 21 and 35 days p.t.i. (e.i. 7 days after the first and third dose, respectively) (n = 5 per group). Spleens were aseptically removed and splenocytes were purified and counted. 5 × 10^5^ cells were stimulated with 100 ng of LPS from *E. coli* (Invivogen) for 24 h. Supernatant were collected and stored at −80°C until analyzed. Cytokine production was determined using commercial ELISA kits (R&D Systems) for mouse TNF, IL-1β and IL-6.

In another experiment, five A20 lymphoma-bearing mice treated with *Leishmania* lysates (L.braz-treated, n = 2, and PD1+L.braz treated, n = 3) and complete recovered were sacrificed at the end of the follow-up period. Five age-matched naive mice served as controls. Spleens were removed, and purified splenocytes were stimulated with 5 μg/mL of A20 cell lysate to recall anti-tumor activity. After 48 h of stimulation, cytotoxicity was assessed by flow cytometry. Briefly, unlabeled splenocytes were used as effectors (E) and A20 lymphoma cells served as targets (T). Tumor cells were labeled with CFSE (Sigma) and seeded into 48-well plates (Nunc) at 2.5 × 10^4^ cells per well. Re-stimulated splenocytes were seeded in 200:1, 100:1 and 25:1 E:T ratios in supplemented RPMI. In parallel, target CFSE-labelled A20 cells were incubated alone to measure spontaneous cell death. Co-cultures were incubated for 18 h, and then cells were collected and stained with propidium iodide (Sigma) prior to analysis by flow cytometry. The percentage of tumor cell death (cytotoxicity) was calculated as: % Cytotoxicity = [(% target cell death - % spontaneous target cell death)/(100 - % spontaneous cell death)] x100.

In another experiment, eight A20 lymphoma-bearing mice treated with 3 doses of *L. braziliensis* lysates (39 days p.t.i.), eight control A20 lymphoma-bearing mice and two age-matched naive mice were sacrificed. Spleens were removed, and cytotoxic assay was performed as described above.

#### Serum proteomics

Serum was obtained from whole peripheral blood collected 35 days post tumor from the PBS- and *L. braziliensis*-treated groups. Targeted proteomics was performed by Proceek © Multiplex Proximity extension assay using the commercially available Olink Proteomics AB (Uppsala Sweden) Mouse Explanatory panel (92 proteins). Expression levels were calculated as described by Koeken et al.^58^ assessing the fold change in between the two groups.

### QUANTIFICATION AND STATISTICAL ANALYSIS

Differences were analyzed using Student’s t test or Wilcoxon signed-rank test. For cytokine production before and after *Leishmania* training, the data are shown as fold increases or raw cytokine compared to the RPMI control. The statistical analyses were performed on these ratios. Analyses were performed using Prism software version 6.0 (GraphPad, San Diego, CA, USA) and R version 4.0.3 (RStudio, Boston, MA, USA). Significance was established as p < 0.05.

## Supplementary Material

dos Santos et al. Supplemental Data

## Figures and Tables

**Table:** KEY RESOURCES TABLE

REAGENT or RESOURCE	SOURCE	IDENTIFIER
Antibodies		
Rabbit polyclonal anti-H3K4me3	Diagenode	Cat#pab-003-050, C15410003-50; RRID: AB_2616052
anti-mouse Lineage Cocktail Pacific Blue	Biolegend	Cat#133310; RRID: AB_11150779
anti-mouse Lineage Cocktail APC	BD Biosciences	Cat#51-9003632
anti-mouse CD117 (ckit) PE	BD Biosciences	Cat#553355; RRID: AB_394806
anti-mouse Ly-6A/E (sca1) PE/Cy7	Biolegend	Cat#122514; RRID: AB_756199
anti-mouse CD34 FITC	eBioscience	Cat#11-0341-85; RRID: AB_465022
anti-mouse CD16/32 BV421	Biolegend	Cat#101332; RRID: AB_2650889
anti-mouse CD135 (FLT3) APC	Biolegend	Cat#135310; RRID: AB_2107050
anti-mouse CD150 PerCP/Py5.5	Biolegend	Cat#115922; RRID: AB_230363
anti-mouse CD48 APC/Cy7	Biolegend	Cat#103432; RRID: AB_1561919
anti-mouse CD45 PE	Biolegend	Cat#103106; RRID: AB_31271
anti-mouse CD11b FITC	Biolegend	Cat#101206; RRID: AB_ 312788
anti-mouse CD8 APC	Biolegend	Cat#100712; RRID: AB_312750
anti-mouse Ly6G BV510	Biolegend	Cat#127633; RRID: AB_2562937
anti-mouse F4/80 APC	Biolegend	Cat#157306; RRID: AB_2832548
biotin anti-PDL1	eBioscience	Cat#13598282; RRID: AB_466837
anti-human TLR2	Invivogen	Cat#maba2-htlr2-2; RRID: AB_11142484
Human Dectin-1/CLEC7A Allophycocyanin Mab	Bio-Techne/R&D	Cat#MAB1859; RRID: AB_2081791
Human Dectin-2/CLEC6A Antibody	Bio-Techne/R&D	Cat#MAB3114; RRID: AB_2081647
anti-hIntegrin beta2 - hIntegrin b2 Affinity Purified Goat IgG	Bio-Techne/R&D	Cat#AF1730; RRID: AB_354957
Normal Goat IgG Control	Bio-Techne/R&D	Cat#AB-108-C; RRID: AB_354267
DC-SIGN Monoclonal Antibody	Bio-Techne/R&D	Cat#MA1-25615; RRID: AB_779638
Mouse IgG2B Isotype Control	Bio-Techne/R&D	Cat#MAB004; RRID: AB_357346
IgG1 Isotype Control	Bio-Techne/R&D	Cat#MAB002; RRID: AB_357344
Human MMR/CD206 Antibody	Bio-Techne/R&D	Cat#AF2534; RRID: AB_2063019
Anti-hMincle-IgG	Invivogen	Cat#mabg-hmcl
InVivoMAb anti-mouse PD-1 (CD279)	BioXcell	Cat#BE0146; RRID: AB_10949053
InVivoMab rat IgG2a isotype control, anti-trinitrophenol	BioXcell	Cat#BE0089; RRID: 1107769
Chemicals, peptides, and recombinant proteins		
β-glucan (β1,3-(D)-glucan	Professor David Williams, College of Medicine, Johnson City, USA	N/A
Lipopolysaccharide	Sigma-Aldrich	Cat#L2880From E. coli serotype 055:B5,
Grace’s insect medium	Gibco, Life Technologies	Cat#11595-030
Heat-inactivated fetal bovine serum (FBS)	Gibco, Life Technologies	Cat#10500064
Protease inhibitor cocktail	Sigma-Aldrich	Cat#P8465
Giemsa	Merck Millipore	Cat#1.09204.0100
Percoll	Sigma-Aldrich	Cat#P1644
Ficoll-Paque	GE Healthcare	Cat#17-1440-03
Roswell Park Memorial Institute medium (RPMI)	Invitrogen	Cat#22406031
TRIzol reagent	Life Technologies	Cat#15596018
16% Formaldehyde	Fisher Scientific	Cat#28908, 11835835
Zymosan	Sigma-Aldrich	Cat#Z4250
Pam3Cys	EMC microcollections	Cat#L2000
Adenosine 5’-triphosphate (ATP) disodium salt hydrate	Merck	Cat#A1852-1VL
2-deoxy-D-glucose	Merck	Cat#D6134
Oligomycin A	Merck	Cat#75351-5MG
FCCP	Merck	Cat#C2920-10mg
Antimycin	Merck	Cat#A8674-25mg
Rotenone	Merck	Cat#R8875-1G
cytochalasin B	Merck	Cat#C6762
Ponatinib	Selleck	Cat#S1490
GW5074 Raf1 kinase inhibitor	Merck	Cat#G6416
SP600125 JNK Inhibitor	Invivogen	Cat#tlrl-sp60
Wortmannin	Invivogen	Cat#tlrl-u0126-5
Torin 1	Invivogen	Cat#inh-tor1
Rapamycin	LC laboratories	Cat#R-5000
R406	EMD Millipore	Cat#505819
luminol (5-amino-2,3, dihydro-1,4-phtalazinedione)	Merck	Cat#521-31-3
SYBR Green	Applied Biosciences	Cat#4368708
APC streptavidin	BD Biosciences	Cat#554067
Phenyl-Sepharose CL-4B	VWR	Cat#17-0810-01
Critical commercial assays		
Pierce BCA protein assay kit	ThermoFisher Scientific	Cat#23225
Human TNFα ELISA	R&D systems	Cat#DY210
Human IL-6 ELISA	R&D systems	Cat#DY206
Human IL-10 ELISA	R&D systems	Cat#DY217B
Human IL-1β ELISA	R&D systems	Cat#DY201
Human IL-1Ra ELISA	R&D systems	Cat#DY280
Lactate Fluorometric Assay kit	Biovision	Cat#K607
iScript cDNA Synthesis Kit	Bio-Rad	Cat#1708891
miRNeasy Micro Kit	QIAGEN	Cat#217084
MACS pan monocyte isolation kit	Milteney Biotec	Cat#130-096-537
QuantSeq 3′ mRNA-Seq Library Prep Kit FWD	Lexogen	Cat#191-196
MinElute PCR purification Kit	QIAGEN	Cat#28006
Max Deluxe set mouse TNFa ELISA	Biolegend	Cat # 430916
Max Deluxe set mouse TNFa ELISA IL6	Biolegend	Cat # 431315
Max Deluxe set mouse TNFa ELISA IL1b	Biolegend	Cat# 43261
Mouse Explanatory panel	Olink Proteomics	Cat#95380
Deposited data		
Raw and analyzed data	This paper	GSE211212
Experimental models: organisms/strains		
*Leishmania Viannia braziliensis*	Leisbank IPTSP/UFG	MHOM/BR/2003/IMG
*Leishmania Viannia guyanensis*	Leisbank IPTSP/UFG	MHOM/BR/2006/PLR6
*Leishmania Leishmania amazonensis*	Leisbank IPTSP/UFG	IFLA/BR/67/PH8
*BALB/c*	Dilave, Uruguay	N/A
C57Bl/6	Dilave, Uruguay	N/A
A20 cell line	ATCC	Cat#TIB-208
B16F10	ATCC	Cat#CRL-6475
Software and algorithms		
GraphPad Prism	Graphpad Software	https://www.graphpad.com
R statistical programming	N/A	RRID:SCR_001905
DESeq2	N/A	RRID:SCR_015687
flowCore	N/A	RRID:SCR_002205
TopSpin 4.0.9	Bruker	https://www.bruker.com
Diva	BD FACSDiva Software v.6.1.3	Cat#643629

## Highlights

- *Leishmania braziliensis* induces trained immunity (TI) in human monocytes
- The secondary stimulus dictates the effector responses produced by trained monocytes
- *L. braziliensis* reduces tumor growth and improves survival in solid tumor models
- *L. braziliensis* impacts the myeloid and T cell accumulation in the tumor environment
