# Supplementary material for: Leishmania braziliensis enhances monocyte responses to promote anti-tumor activity
Source: Cell Rep. Author manuscript; Available in PMC 2024 Apr 8. (PMC11000460; doi:10.1016/j.celrep.2024.113932)
Supplement: dos Santos et al. Supplemental Data [file NIHMS1978493-supplement-dos_Santos_et_al__Supplemental_Data.pdf]

Cell Reports, Volume *43*

Supplemental information

Leishmania braziliensis enhances monocyte responses to promote anti-tumor activity

Jéssica Cristina dos Santos, María Moreno, Lisa U. Teufel, Sofía Chilibroste, Samuel T. Keating, Laszlo Groh, Jorge Domínguez-Andrés, David L. Williams, Zuchao Ma, Douglas W. Lowman, Harry E. Ensley, Boris Novakovic, Fátima Ribeiro-Dias, Mihai G. Netea, José A. Chabalgoity, and Leo A.B. Joosten

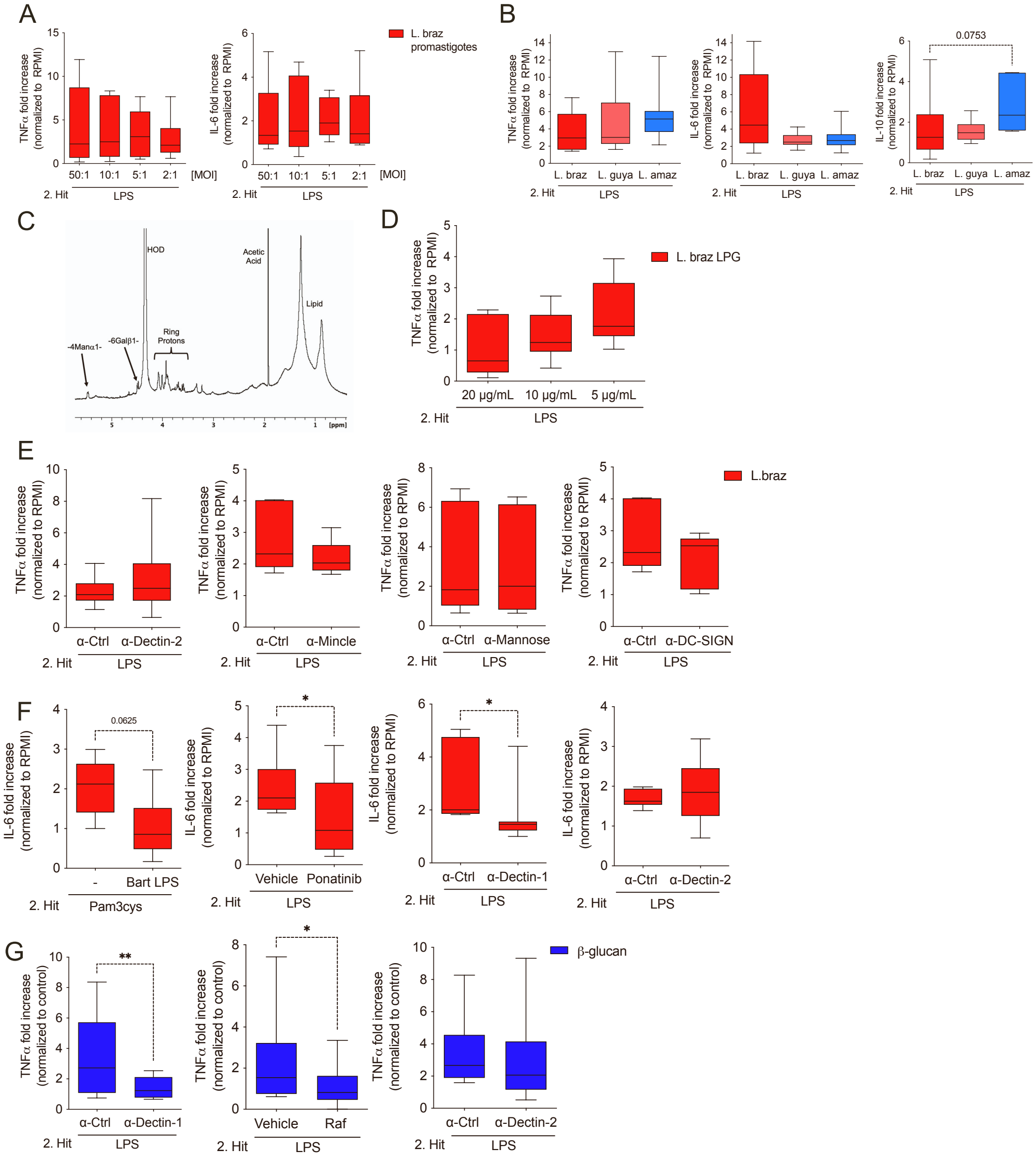

Figure S1. ***Leishmania spp*. induces trained immunity, Related to Figure 1.** (**A**) TNF and IL-6 release from monocytes trained with RPMI, or *L. braziliensis* live promastigotes (MOI 50, 10, 5 and 1) for 24 hours after LPS (10 ng/ml) restimulation at day 7, measured by ELISA. (**B**) TNF, IL-6 and IL-10 release from *L. braziliensis*. *L. guyanensis* and *L. amazonensis* lysates-trained macrophages (25 µg/mL) after restimulation with LPS (10 ng/mL) at day 7. (**C**) Characterization of lypophosphoglycan (LPG) fraction of *L. braziliensis* by 1D ^{1}H NMR analysis at 60°C in D_2O. (**D**) TNF production after LPS restimulation at day 7 of *L. braziliensis*-LPG-trained (20 µg/mL, 10 µg/mL, 5 µg/mL) macrophages. (**E**) TNF production after LPS restimulation at day 7 of *L. braziliensis*-trained (25 µg/mL) macrophages ± anti-Dectin-2, anti-Mincle, anti-Mannose and anti-DC-SIGN. (**F**) IL-6 production after LPS or Pam3Cys restimulation at day 7 of *L. braziliensis*-trained (25 µg/mL) macrophages ± *B. quintana* LPS (Bart. LPS, TLR4 antagonist), the RIP2 kinase inhibitor ponatinib, anti-Dectin-1, anti-Dectin-2. (**G**) TNF production after LPS restimulation at day 7 of β-glucan-trained (5 µg/mL) macrophages ± anti-Dectin-1, Raf inhibitor GW5074, and anti-Dectin-2. *n*= 6 independent donors. Cytokine measurements are represented as fold increase normalized to RPMI

(non-trained cells). The data is shown in box and whiskers (Min to Max) plots from 2 independent experiments (*$p < 0.05$ by Wilcoxon test).

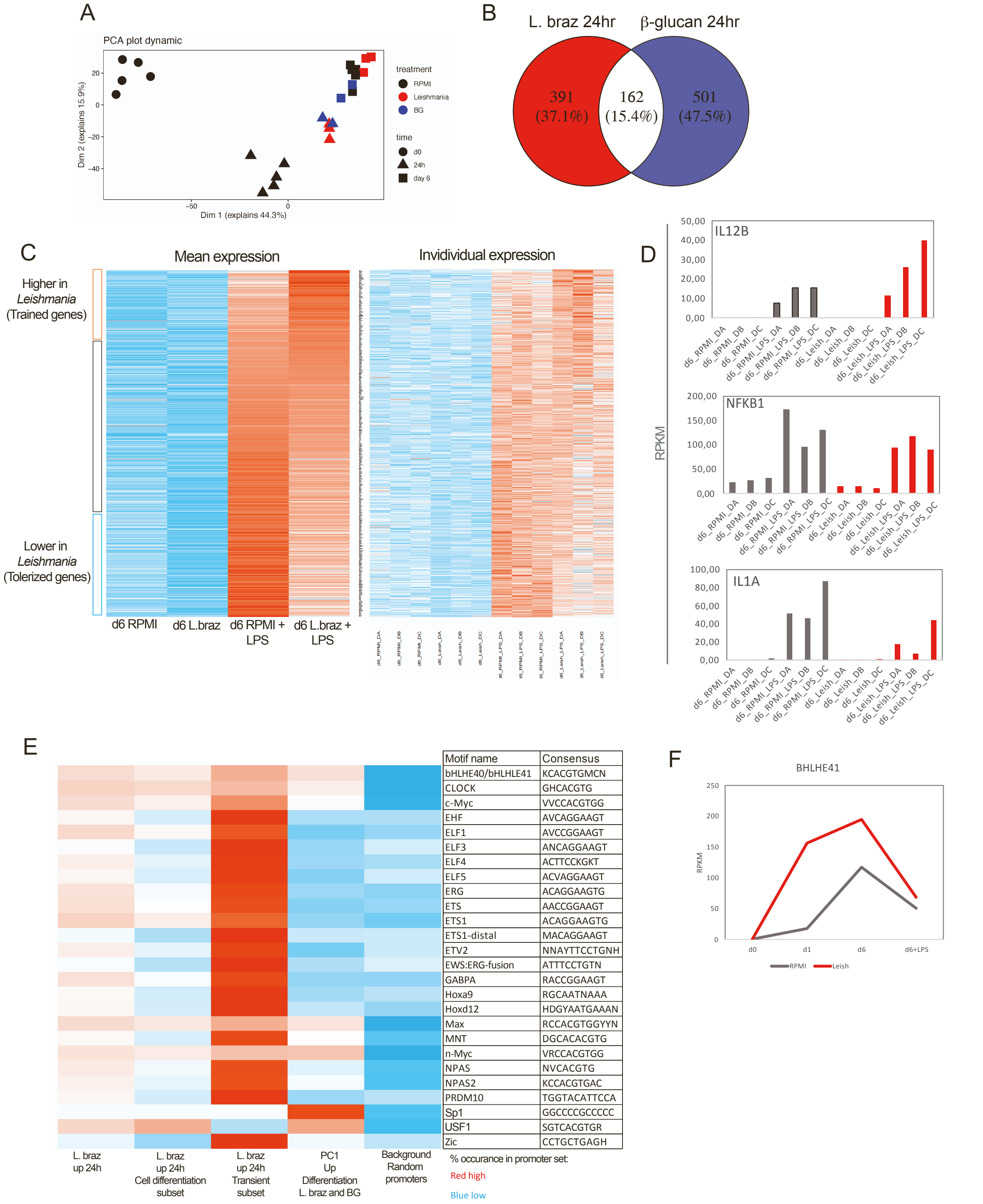

Figure S2. Transcriptional responses induced by *L. braziliensis* and β-glucan upon training, Related to figure 1. (**A**) Principal component plot (PCA) of gene expression dynamic across treatment and time

points (RPMI, black; *L. braziliensis* lysates, red; β-glucan, blue; day 0, circle; 24 hours, triangle; day 6, square). (**B**) Venn diagram representing the percentage of modulated genes which are *L. braziliensis*-, β-glucan-specific or common to both stimuli after 24-hours exposure. (**C**) Heatmap showing the mean and individual gene expression (upregulated, red; downregulated, blue) at the indicated time points (day 6) after RPMI or *L. braziliensis* lysates exposure (25 μg/mL) followed by LPS restimulation (10 ng/mL). (**D**) Example of genes which were either upregulated, equal, or downregulated in *L. braziliensis*-trained cells restimulated with LPS. (**E**) Transcription factors motif analysis performed in the subset of genes which were transient or associate with cell differentiation processes after 24 hours exposure with *L. braziliensis*. (**F**) Time-resolved median expression of *BHLHE41*, which is one of the TF associated with the transient subset of upregulated genes in *L. braziliensis*-exposed cells. *n*= 3 independent donors.

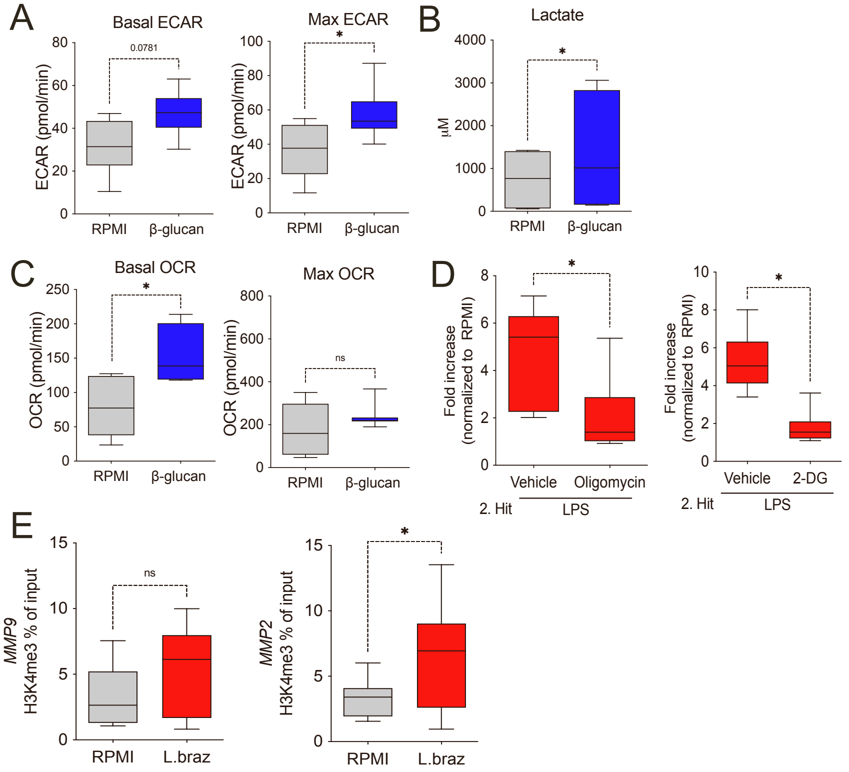

Figure S3. **Metabolic routes involved in trained immunity, Related of Figure 2.** (**A**) Basal and maximum extracellular acidification rates (ECAR) of RPMI- and β-glucan-trained macrophages at day 6, measured by seahorse. (**B**) Lactate production assessed in the supernatant of trained macrophages at day 6 by fluorometric assay. (**C**) Basal and maximum oxygen consumption rate (OCR) of RPMI- and β-glucan -trained macrophages at day 6, measured by seahorse. (**D**) TNF production after LPS restimulation at day 7 of *L. braziliensis*-trained (25 μg/mL) macrophages ± the OXPHOS and glycolysis inhibitors 2-DG and oligomycin, respectively. (**E**) Levels of H3K4me3 at *MMP9* and *MMP2* promoters of RPMI- and *L. braziliensis*-exposed macrophages assessed by ChiP-qPCR. *n*= 6 independent donors. Cytokine measurements are represented as fold increase normalized to RPMI (non-trained cells). The data is shown in box and whiskers (Min to Max) plots from 2 independent experiments (*$p < 0.05$ by Wilcoxon test; ns $p > 0.05$).

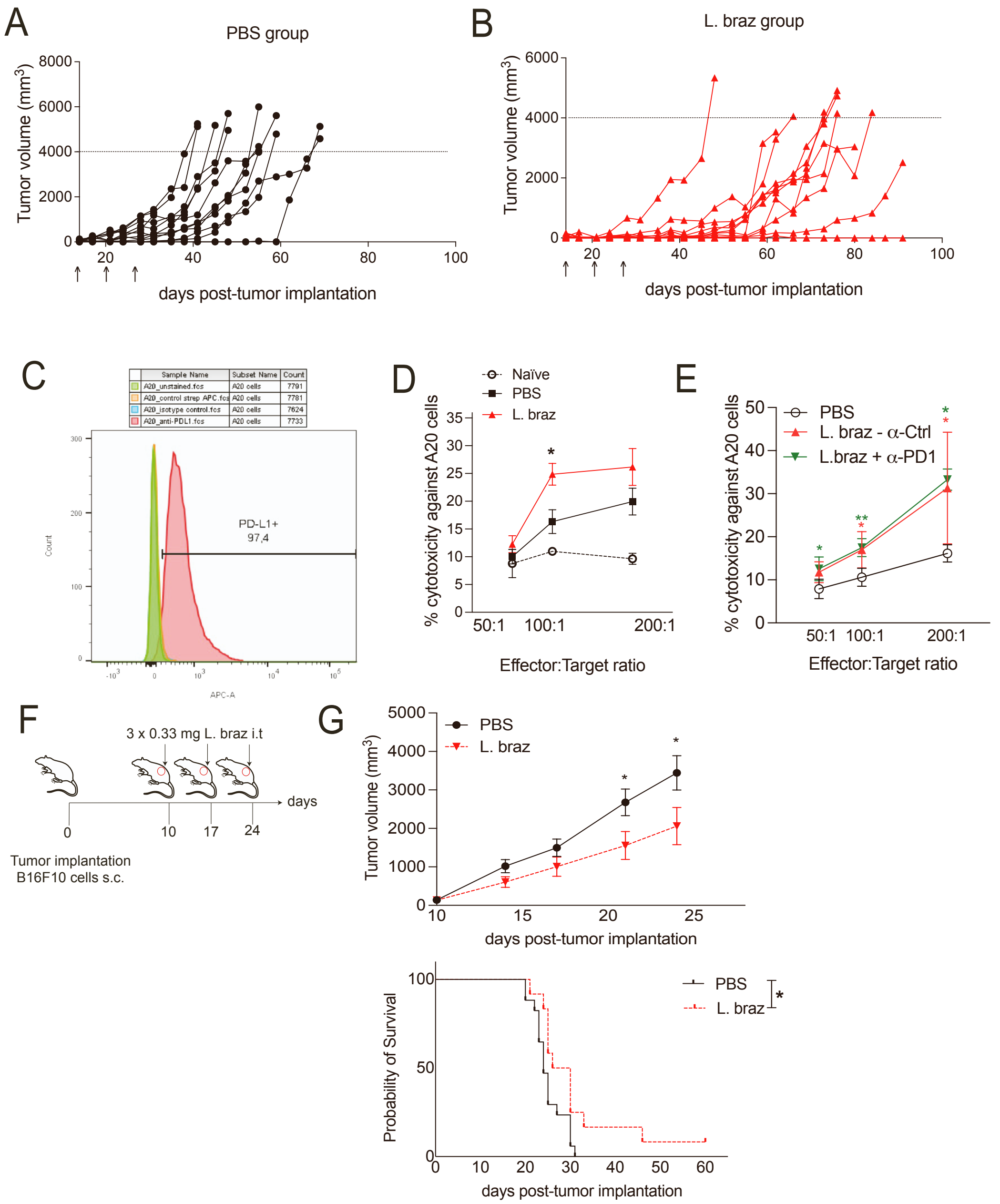

Figure S4. ***L. braziliensis* exerts anti-tumor activities *in vivo*, Related to Figure 4.** (**A-B**) *In vivo* tumor growth curves of showing the tumor volume of each BALB/c mouse individually. Mice were inoculated with 1×10^6 A20 tumor cells and submitted to a regimen consisted of three subcutaneously injections of PBS- or *L. braziliensis* lysates (0.33 mg/mL each injection). Overall survival was followed up for 100 days (n = 12). Significance was calculated in between the groups (*p < 0.05 by t-test). (**C**) PDL-1 expression in the A20 cells assessed by flow cytometry. (**D**) Cell-mediated cytotoxicity percentage at different ratios E:T performed in naïve, PBS, *L. braziliensis*-treated mice 39 days post-tumor implantation (n=8). (**E**) Cell-mediated cytotoxicity percentage at different ratios E:T performed in *L. braziliensis*-anti-PD1 and *L. braziliensis*-isotype control-treated mice which survived until the end of the follow up period (n = 12). Splenocytes were used as effector cells and A20 as target cells. (**F-G**) C57BL6 mice were inoculated with 2.5×10^5 B16F10 tumor cells and submitted to a regimen consisted of three intratumorally injections of PBS- or *L. braziliensis* lysates (0.33 mg/mL each injection). Tumor growth and survival was followed up for 60 days (n = 12). Significance was calculated in between the groups (*p < 0.05 by *t*-test).

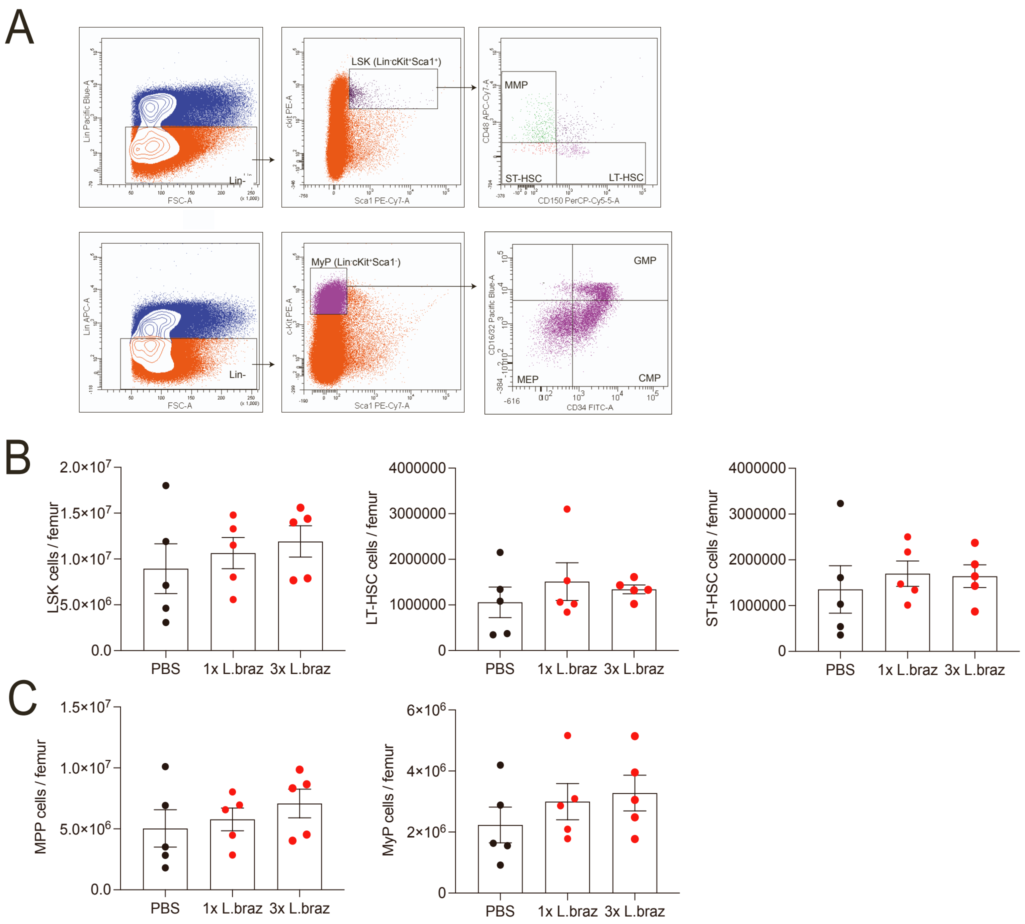

Figure S5. **Bone marrow effects induced by *L. braziliensis* exposure *in vivo*, Related to Figure 4.** (**A**) Representative flow cytometry plots of bone marrow phenotyping. Mice were injected with one single dose of *L. braziliensis* for 7 days or three doses of *L. braziliensis* or PBS every 7 days. Bone marrow cells were collected for *ex vivo* immunophenotyping analysis by flow cytometry. (**B**) Absolute numbers of LSKs, LT- and ST-HSCs, (**C**) MMPs, and MyPs in PBS- and *L. braziliensis*-treated mice (n=5). The data is shown in bar plots (Mean ± SD).

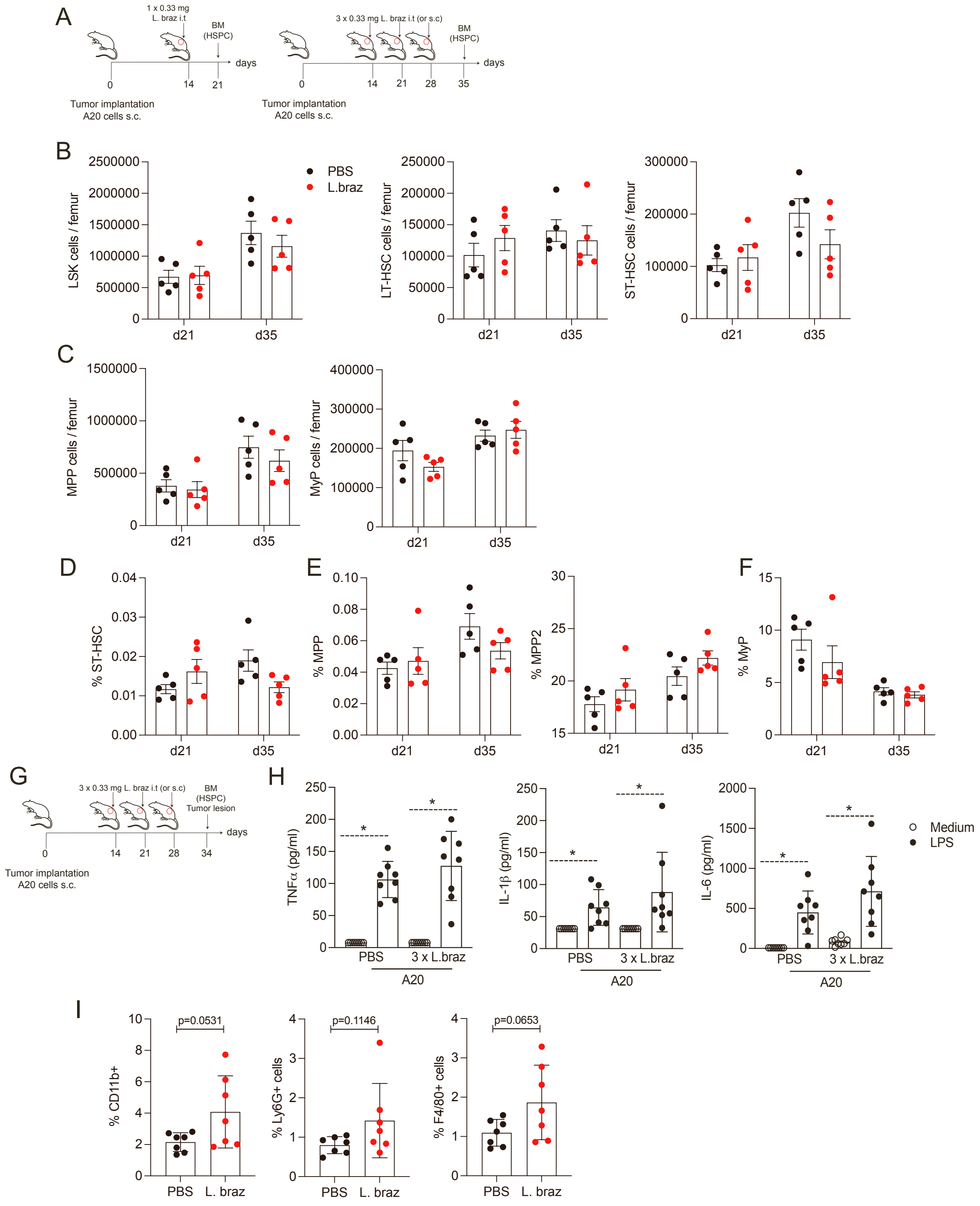

Figure S6. **Post-tumor effects induced by *L. braziliensis* at the bone marrow and at the tumor site, Related to Figure 5**. (**A**) Experimental setup. *Ex vivo* bone marrow phenotyping of tumor-bearing PBS- and *L. braziliensis*-treated (0.33 mg/mL) mice submitted to both single (day 21) and triple (day 35) doses regimen injected subcutaneously. (**B**) Absolute numbers of LSKs, LT- and ST-HSCs, (**C**) MMPs and MyPs, (**D**) percentages of ST-HSCs, (**E**) MMPs, MMP2 and (**F**) MyPs assessed by flow cytometry (n=5). (**G**) Cytokine production of bone marrow cells and innate immune cells infiltration of tumor-bearing PBS- and *L. braziliensis*-treated (0.33 mg/mL) mice submitted to triple (day 34) doses regimen injected subcutaneously. (**F**) 1×10^5 bone marrow cells were restimulated for 24 hours with LPS (10 ng/mL). (**H-I**)

TNF, IL-1β and IL-6 production was assessed in the supernatant by ELISA (n=8). Percentages $CD11b^+$, $Ly6G^+$ and $F4/80^+$ cells into the tumor lesion were assessed by flow cytometry (n=7). The data is shown in bar plots (Mean ± SD).
